# OVCH1 Antisense RNA 1 is differentially expressed between non-frail and frail old adults

**DOI:** 10.1007/s11357-023-00961-9

**Published:** 2023-10-11

**Authors:** Imad Abugessaisa, Ri-Ichiroh Manabe, Tsugumi Kawashima, Michihira Tagami, Chitose Takahashi, Yasushi Okazaki, Stefania Bandinelli, Takeya Kasukawa, Luigi Ferrucci

**Affiliations:** 1 Laboratory for Large-Scale Biomedical Data Technology, RIKEN Center for Integrative Medical Sciences, 1-7-22 Suehiro-cho, Tsurumi-ku, Yokohama City, Kanagawa 230-0045 Japan; 2 Laboratory for Comprehensive Genomic Analysis, RIKEN Center for Integrative Medical Sciences, 1-7-22 Suehiro-cho, Tsurumi-ku, Yokohama City, Kanagawa 230-0045 Japan; 3 Azienda USL Toscana Centro, InCHIANTI, Villa Margherita, Primo piano Viale Michelangelo, 41, 50125 Firenze, Italy; 4 Institute for Protein Research, Osaka University, Suita, Osaka, 565-0871 Japan; 5 National Institute on Aging, National Institutes of Health, MedStar Harbor Hospital 5th floor, 3001 S. Hanover Street, Baltimore, MD 21225 USA

**Keywords:** Frailty, CAGE, GWAS-LD enrichment, Transcription start site (TSS), Promoters, Enhancers, Motif activities, OVCH1 Antisense RNA 1, Glycogen Phosphorylase L

## Abstract

While some old adults stay healthy and non-frail up to late in life, others experience multimorbidity and frailty often accompanied by a pro-inflammatory state. The underlying molecular mechanisms for those differences are still obscure. Here, we used gene expression analysis to understand the molecular underpinning between non-frail and frail individuals in old age. Twenty-four adults (50% non-frail and 50% frail) from InCHIANTI study were included. Total RNA extracted from whole blood was analyzed by Cap Analysis of Gene Expression (CAGE). CAGE identified transcription start site (TSS) and active enhancer regions. We identified a set of differentially expressed (DE) TSS and enhancer between non-frail and frail and male and female participants. Several DE TSSs were annotated as lncRNA (XIST and TTTY14) and antisense RNAs (ZFX-AS1 and OVCH1 Antisense RNA 1). The promoter region chr6:366,786,54-366,787,97;+ was DE and overlapping the longevity CDKN1A gene. GWAS-LD enrichment analysis identifies overlapping LD-blocks with the DE regions with reported traits in GWAS catalog (isovolumetric relaxation time and urinary tract infection frequency). Furthermore, we used weighted gene co-expression network analysis (WGCNA) to identify changes of gene expression associated with clinical traits and identify key gene modules. We performed functional enrichment analysis of the gene modules with significant trait/module correlation. One gene module is showing a very distinct pattern in hub genes. Glycogen Phosphorylase L (PYGL) was the top ranked hub gene between non-frail and frail. We predicted transcription factor binding sites (TFBS) and motif activity. TF involved in age-related pathways (e.g., FOXO3 and MYC) shows different expression patterns between non-frail and frail participants. Expanding the study of OVCH1 Antisense RNA 1 and PYGL may help understand the mechanisms leading to loss of homeostasis that ultimately causes frailty.

**Supplementary Information:**

The online version contains supplementary material available at 10.1007/s11357-023-00961-9.

## Introduction

Increasing age is associated with increasing prevalence of frailty, a condition that is strongly associated with multimorbidity, disability and high risk of hospitalization, falls, and short-term mortality [1]. However, the biological mechanisms that causes loss of homeostasis and ultimately frailty are not understood [2, 3]. Elucidating these mechanisms is essential to develop new diagnostic tools that allow the identification of frailty at an early stage so that interventions can be implemented that reduce the risk of overt frailty and prevent its consequences. It has been suggested that the biological mechanisms of aging are at the root of frailty. However, despite intense research and few conceptual and empirical breakthroughs, our understanding of the mechanisms of aging remains substantially limited.

In this study, we aim to contribute to the current research and understating of frailty in old human adults performing Cap Analysis of Gene Expression (CAGE) analysis of whole-blood samples collected from 12 non-frail and 12 frail individuals who were participants of a longitudinal study of aging. We performed two sequencing assays, no-amplification non-tagging CAGE (nAnT-iCAGE) [4] and Low Quantity Single Strand CAGE (LQ-ssCAGE) [5]. Both assays are version of the CAGE method which capture the 5′-end of messenger RNA. CAGE provides information on two aspects of the capped transcriptome. First, it provides genome-wide single base-pair resolution map of transcriptional start site (TSS). Secondly, it quantifies the level of transcripts initiated at each TSS. CAGE profiles enable 5′-end expression profiling, studying promoter architecture and estimation of the activity of enhancers [6]. CAGE profiles can also help predicting transcription factor binding site (TFBS) and perform motif activity analysis. To enhance our understanding of the CAGE data, we used DESeq2 [7] and weighted gene co-expression network analysis (WGCNA) [8] to perform differential expression analysis of the identified TSSs and enhancers. WGCNA creates modules (sets of genes) based on the CAGE expression profiles and relates them to clinical traits. WGCNA allows enrichment analysis using different databases of reference (Reactome, KEGG, MSigDB, gene ontology, etc.) to make inference of what biological mechanisms are different between the pre-defined phenogroups (non-frail and frail).

We utilized the GWAS-LD enrichment analysis tool (https://reftss.riken.jp/reftss/GWAS-LD_enrichment) to find DE TSS and enhancer regions overlapping LD blocks on the genome. GWAS-LD enrichment analysis enabled us to identify GWAS traits overlapping the DE regions.

We hypothesized that the biological mechanisms of aging involve the fine turning of gene expression through the modulation of gene’s promoters and enhancers. To test this hypothesis, we analyzed 5′-end gene expression profiles and studied their association with frailty and clinical phenotypes using blood samples and clinical data collected in the InCHIANTI study [9].

## Methods

### Participants and InCHIANTI study

The study subjects (*n*=24) were selected among the participants of the InCHIANTI epidemiologic study [9]. InCHIANTI is a representative population-based study of older persons living in the Chianti geographic area (Tuscany, Italy). The main research focus of InCHIANTI was the study of risk factors of mobility disability in old age. All participants responded to an interview that focused on information on medical history, life habits, and level of physical activity, and most of them donated a blood sample that was collected in the morning after an overnight fasting. A medical exam was performed within a week from the interview that included a full medical and functional examination, several physical performance tests, and a mini-mental state examination (MMSE). The study started in 1998 (baseline) and was followed by 4 follow-ups. In our current study, we selected participants in the fourth follow-up who were aged > 60 years and had complete data on clinical and functional evaluation. Controls (non-frail group) had no major chronic diseases, no overt cognitive impairment (MMSE>24), walking speed >1.0 m/sec, and not self-reported disability in activities of daily living. The diagnosis of frailty was established based on the positivity of at least three over 5 criteria, namely, “unintentional weight loss,” “weakness,” “lack of energy,” “slowness,” and “sedentariness” proposed by Fried and colleagues [1]. The assessment of the frailty criteria in the InCHIANTI study has been reported elsewhere [10].

Participants’ characteristics are summarized in Table 1 and described in [9]. The InCHIANTI study protocol and consent form were approved by the local ethical committee of the USL 10 in Florence Piero Palagi Hospital, Villa Margherita Viale Michelangelo, 41 50125 Florence, according to the principles of the Declaration of Helsinki, and all participants signed a written consent. A *t*-test of clinical variables in Table 1 shows that some of the clinical traits were significantly different between non-frail and frail (frailty differences) and male and female (sex differences).

**Table 1:** Study participants’ characteristics (data presented as mean ± SD). *P*-value obtained using Welch’s *t*-test.

Variables	Total sample	Non-frail (5 M/7 F)	Frail (6 M/6 F)	*P*-value *t*-test (Frailty)	*P*-value *t*-test (Sex)
Age (year)	84.8 ± 3.9	83.2 ± 4.3	86.4 ± 2.8	0.04	0.04
Weight (kg)	69.4 ± 16.9	70.4 ± 18.05	68. 4± 16.4	ns	0.02
Height (cm)	154.4 ± 11.1	157.3 ± 9.21	151.5 ± 12.5	ns	< 0.001
HDL cholesterol (mg/dL)	51.5 ±18.7	56.4 ±21.3	46.5 ±14.9	ns	0.06
Triglycerides (mg/dL)	135 ± 56.6	138.5 ± 56.01	131.5 ± 59.4	ns	ns
Red blood cells (RBC) (*n*, millions/μL)	4.5 ± 0.44	4.7 ± 0.33	4.4 ± 0.51	ns	ns
Hemoglobin (g/dL)	13.3 ± 1.67	12.7 ± 1.23	12.7 ± 1.86	0.06	ns
Hematocrit (%)	41.2 ± 4.39	42.8 ± 2.9	39.6 ± 5.1	ns	ns
Blood glucose (mg/dL)	108.7 ± 37.2	118.1 ± 48.7	99.25 ± 18.22	ns	ns
Blood urea nitrogen (mg/dL)	46.08 ± 20.3	44.2 ± 10.6	47.9 ± 27.3	ns	ns
Serum creatinine (mg/dL)	0.96 ± 0.50	0.89 ± 0.28	1.03 ± 0.6	ns	0.03

### Total RNA extraction and RNA sequencing library preparation

**Fig. 1:**
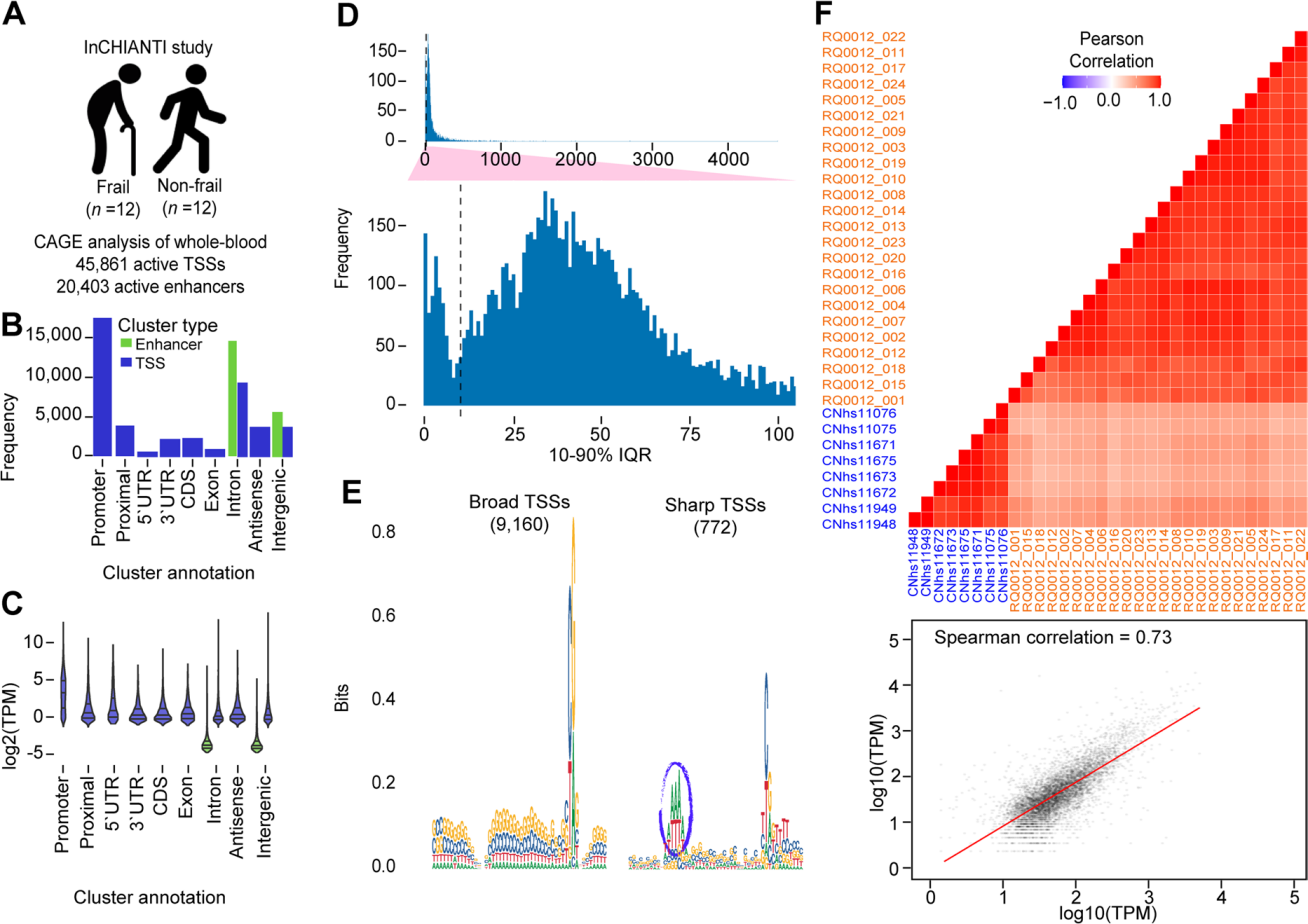
Defining enhancers and TSS landscape InCHIANTI study (*N*=24). **A** Overview of data set. Basic demographic information, frailty data, global cognitive performance, medical laboratory test results, and whole-blood samples were taken from InCHIANTI study. Based on frailty criteria, subjects are classified into non-frail (*N*=12) and frail (*N*=12). For each subject, a CAGE library was produced (24 libraries); CAGE analysis enables the mapping of TSSs (45,861) and enhancer (20,403) regions. **B** Number of clusters within each annotation category. The detected TSS and enhancers were merged and produced merged clusters (65,441) clusters. **C** Expression of clusters within each annotation category. **D** Bimodal distribution of interquartile ranges (IQRs) of highly expressed TSSs; the interquartile range (IQR) can be used to find sharp and broad TSSs. The top panel (zoom-in panel) highlights the distinction between the two classes of TSSs. Most TSSs are either below or above 10 bp IQR (the dashed line) which indicated the cutoff to classify TSSs into Sharp and Broad. **E** Sequence logos of core promoter regions of sharp and broad TSSs. Sharp TSSs (right) tend to have a stronger TATA-box (ellipse) upstream of the TSS compared to Broad TSSs (left). **F** Correlation with FANTOM5 whole-blood samples. FATOM5 whole-blood samples profiled with CAGE to map TSS landscape. The top panel shows the correlation matrix of all samples from InCHIANTI (*N*=24) and FANTOM5 whole-blood samples (*N*=8). The bottom panel scatter plotter showing the correlation between library RQ0012_005 and FANTOM5 library CNhs11949

Total RNA was extracted from whole 2.5 -L PAXgene tubes of whole blood following the RNeasy Mini kit according to the manufacturer’s instructions (QIAGEN). We prepared two types of sequencing libraries (Fig. 1A). Preparation of no-amplification non-tagging CAGE (nAnT-iCAGE) and Low Quantity Single Strand CAGE (LQ-ssCAGE) libraries at RIKEN was approved by the institutional review board at the Yokohama Campus (approval number H29-4(3)). Detailed description of library preparation and QC, sequencing, and mapping of raw reads procedures are provided in Supplementary information.

### Generation and post-processing of CAGE transcription start sites

To obtain single-nucleotide TSS, mapped reads in BAM format were postprocessed to obtain CAGE transcription start sites at single-nucleotide resolution [11] in a BED formatted file. Using in-house script, we generated BED files from the mapping results of both nAnT-iCAGE and LQ-ssCAGE libraries. The resulting BED files with TSS genomic coordinates were annotated, quantified, and analyzed as described in the Supplementary information. The resulting CAGE dataset unique quality and characteristics were further checked using a published method that performs quality control on the CAGE profiles (Supplementary Fig. 1 and 2 and Supplementary information).

### Differential expression analysis of TSS and enhancers

The R Bioconductor package DESeq2 [7] was used to perform differential expression analysis (DEA). Before we conducted the DEA, we performed visual inspection to identify outliers. A visual inspection of the expression matrix through the principal component analysis showed no outlier (Supplementary Fig. 3). A DESeq2 object with blank design using the function DESeqDataSet() was created. We used the normalization and log transform standard procedures in DESeq2. Using the available clinical data from InCHIANTI database, we added contrasts to compare the gene expression between non-frail and frail (frailty condition) and male and female (sex) participants. DEA provides us with a set of DE TSS and active enhancer regions to be used for further analysis and interpretation.

### Genomic and functional annotation of the DE TSS and enhancers

Genomic annotation of the DE TSS and enhancer regions assign genomic feature (transcripts, genes, and proteins) to the DE regions. Functional annotation tries to find matching, Gene Ontology (GO) terms, KEGG pathways, diseases, or hits in published articles about the set of genes assigned to the DE TSS and enhancer regions.

To perform the two types of annotation for the DE regions, we utilized two databases. The refTSS is a database with 241,049 curated published TSS peaks on human genomes [12]. Each TSS peak in refTSS has a unique ID (refTSS ID). And each peak is defined with its genomic coordinates (chromosome name, start, and end position of the peak and the strand (+/−)). In refTSS database, TSS peak was annotated to the public gene and protein mode in two steps. First, TSS peak was assigned to the nearest transcripts from public transcript sets (within 500 bp for polII transcripts and 50 bp for non-polII transcripts). In the second step, gene and protein annotations assigned to the nearest transcripts were transferred to the TSS peaks [12].

To find overlapping genomic regions between DE TSS and enhancer regions and refTSS TSS peaks, we used intersectBed command from the BEDTools suite [13]. intersectBed gave the list of refTSS IDs that overlapped with DE TSS and enhancer regions “*overlapped refTSS IDs*.” Next, we obtained the annotation files from the refTSS database (https://reftss.riken.jp/datafiles/current/human/). We used Linux grep command with -f option to pull all annotation for the list of “*overlapped refTSS IDs*.” This enabled us to find associated human genes with “*overlapped refTSS IDs*.” The list of associated genes used as input for functional annotation. We utilized GeneCards suite [14], The Human Protein Atlas [15], and DAVID Bioinformatics Resources [16] for functional annotation.

In addition to the refTSS database, we used the Human Ageing Genomic Resources [17]. We downloaded the set of the GenAge (a list of all human aging genes from the human dataset) (*n*= 307) as of 2023-08-23. We used intersectBed command from BEDTools suite as described above to find the overlap between the DE regions and GeneAge.

### GWAS-LD enrichment analysis of the DE regions

The set of overlapped refTSS IDs was used as input for GWAS-LD analysis to find the number of DE TSS/enhancers overlapping with LD blocks on the genome. LDLink v5.1 [18], and the NHGRI-EBI catalog of human genome-wide association studies v1.0.2 [19] is used as the background dataset for the GWAS-LD enrichment analysis. The GWAS-LD analysis tool was explained in https://reftss.riken.jp/reftss/Manual, in short, the tool search for TSS/enhancer regions overlapping LD block in LDLink v5.1. If the number of overlapping TSSs is significantly higher than the expected frequency by chance it indicates enrichment in LD blocks. GWAS-LD utilizes trait information from the GWAS of SNPs associated with LD blocks to group LD blocks using traits as labels. To test for the significance of the overlapping TSS/enhancer result, GWAS-LD implements a hypergeometric test for each of GWAS-LD groups.

### Weighted gene co-expression network analysis and PPI

To identify modules of correlated genes and find hub genes, we used the R package weighted correlation network analysis (WGCNA) [8]. WGCNA was started by constructing a gene co-expression network [8]. To identify the hub genes in the distinct module from WGCNA, we utilized cytoHubba app in(v3.9) [20]. The computation and analysis of WGCNA is described in the Supplementary information. We performed functional annotation for the set of a module eigengene, with the highest correlation and smaller *P*-value for each clinical trait. DAVID Bioinformatics Resources [16] was used for the functional annotation of the module member genes.

### Enrichment of DNA-binding motifs and motif activity calculation and analysis

After identifying the differential expressed TSSs and active enhancers, we aim to predict the set of the transcription factors (TF) involved in the regulation of the TSSs and active enhancers (Supplementary information). CAGE data enabled motif activity calculation and analysis. The method used to calculate motif activity and analysis was elaborated in the Supplementary information. As described in [21], a (DNA) motif is a unique DNA sequence to which a corresponding group of TF proteins binds to, and motif activity “*represents the time-dependent nuclear activity of positive and negative regulatory factors that bind to the sites of the motif*.” We tested the kind of the probability distribution function that can fit the distribution of motif activity values; we found that Weibull distribution [22] was the good fit.

## Results

### CAGE profiles of non-frail and frail subjects

The CAGE profiling results in 45,861 TSS and 20,403 active enhancers (Fig. 1a and Data Availability). The majority of the TSS clusters from the CAGE profiles were located within gene promoter regions (Fig. 1B). However, novel TSS were also found in other locations categories (proximal, 5′-UTR, 3′-UTR, CDS, etc.) As could have been expected, most of the enhancer clusters were detected within the intron and intergenic regions (Fig. 1B). In addition to the cluster annotation, we looked at the expression of the clusters within each genomic annotation category. Fig. 1C indicates that TSS annotated promoters were highly expressed compared to the other TSSs (novel TSSs). As expected, enhancer expression was lower than TSSs. We analyzed the shape of the TSS to find the sharp and broad promoters [23]; using the highly expressed TSSs with TPM ≥ 10, we calculated the Interquartile Range (IQR) and found 9916 broad promoters and 772 sharp promoters (Fig. 1D). The sequence log of two types of the promoters shows that sharp TSSs tend to have a stronger TATA-box upstream of the TSS compared to broad TSSs (Fig. 1E). Finally, we investigated the correlation between the CAGE profiles with eight libraries generated from human whole-blood samples in the Functional ANnoTation Of the Mammalian genome (FANTOM5) project [11, 24]. Pearson’s correlation coefficients indicate a positive correlation in the expression profiles of the FANTOM5 whole-blood samples and the 24 samples from or study as indicated by the correlation heatmap matrix in (Fig. 1F top). Furthermore, Spearman’s correlation between sample from the current study and sample from FANTOM5 was 0.73 (Fig. 1F bottom).

### Differential expression analysis of TSSs and enhancers

**Fig. 2:**
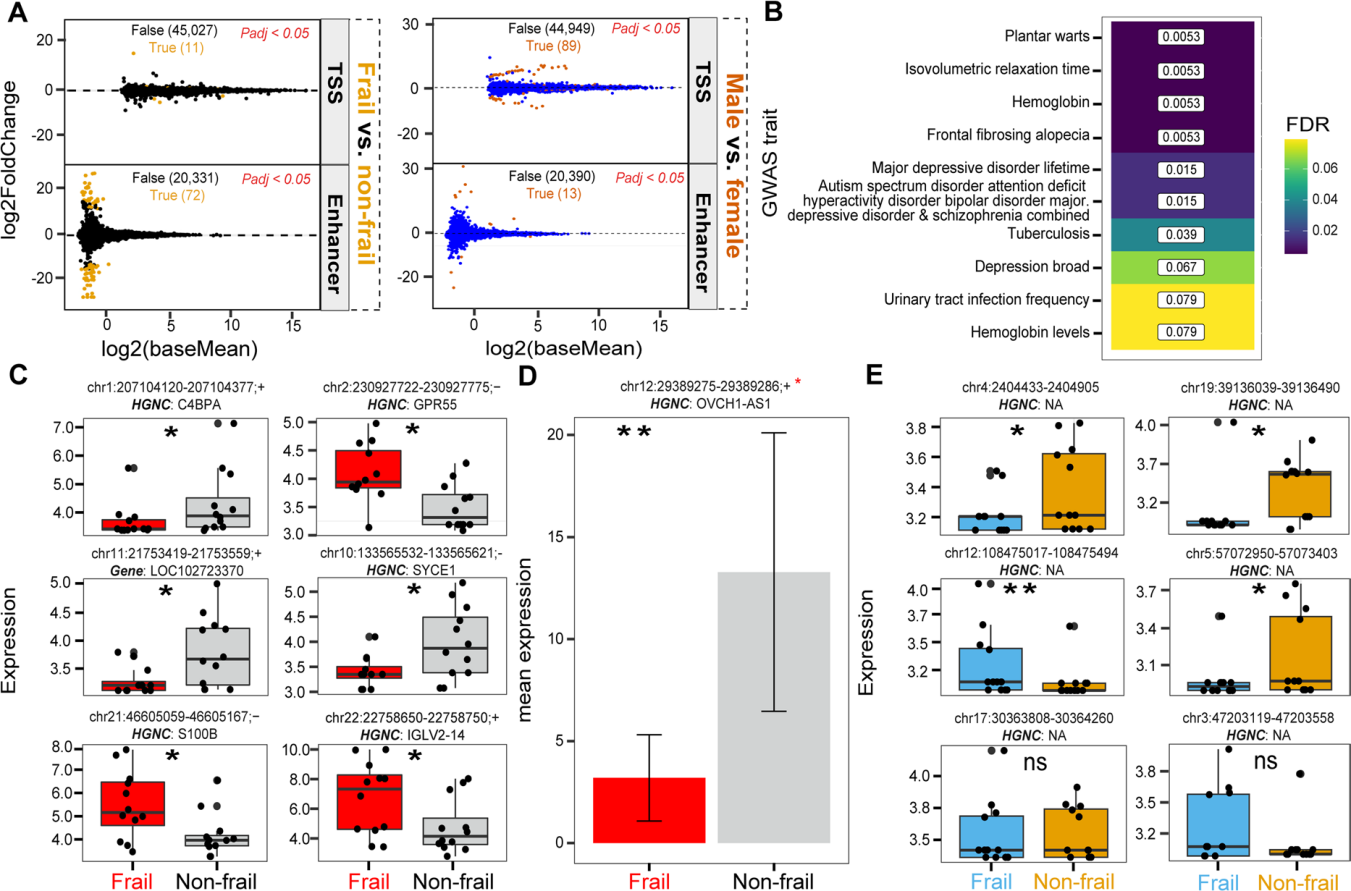
Differential expression analysis of the detected TSS and enhancers. **A** Diagnostic MA plot of the differential expression analysis of TSS and enhancer regions frail vs. non-frail (left) and male vs. female (right). **B** Heatmap of GWAS-LD enrichment analysis of the differentially expressed TSS and enhancer regions. The labels represent the GWAS trait, and the numbers in the cells are the FDR values. **C** Expression profile of top differentially expressed TSS frail vs. non-frail. **D** Mean expression OVCH1-AS1. Error bars represents standard error of the mean. **E** Expression profile of top differentially expressed enhancer frail vs. non-frail. *P < 0.05, **P < 0.01, ***P < 0.001, and ****P < 0.0001; ns, not significant by a two-way ANOVA test

We found a set of TSS and active enhancer regions differentially expressed (DE) between non-frail and frail (frailty) and male and female (sex) participants (**Data Availability**). The DESeq2 analysis result revealed 11:45,207 DE TSSs and 72:20,404 DE active enhancers (*Padj* < 0.05) (Fig. 2A left**)**. The total number of DE regions is 83. When we intersect the 83 DE regions with refTSS database, we found 19 overlapped regions. Likewise, between male and female, we found 89:45,207 DE TSS and 13:20,404 DE active enhancers (*Padj <* 0.05) (Fig. 2A right). The total number of DE regions is 102. The intersect of the 102 DE regions with refTSS database gave 171 overlapped regions.

### Functional annotation and GWAS-LD enrichment analysis of the DE regions

#### Annotation of genes associated with DE regions between non-frail and frail (frailty differences)

The intersection of the DE TSS and active enhancers regions (non-frail vs. frail) with refTSS database resulted in 19 genomic regions. We found four genes associated with 10 regions (9 regions are not associated with any genes) (Supplementary Table 1). OVCH1 Antisense RNA 1 (OVCH1-AS1) is among the associated genes. OVCH1-AS1 is a lncRNA and belongs to the antisense RNAs and has 5 transcripts. Antisense RNAs were found to play a role in regulating gene expression during replication, transcription, and translation [25]. In the NHGRI-EBI Catalog of human GWAS, 10 associations (variant and risk allele) were associated with OVCH1-AS1. Alzheimer disease and age of onset are among the top traits for OVCH1-AS1. The second gene was free fatty acid receptor 2 (FFAR2), a protein coding gene with 3 transcripts. Cell line study found that FFAR2 is expressed in neuronal cells [26]. The study observed that FFAR2 inhibition increases Aβ-Induced neurotoxicity. Using DAVID Bioinformatics tool [16], we found that the cAMP signaling pathway from KEGG was associated with FFAR2 as well as immunity and inflammatory response biological process. Diversion colitis and inflammatory bowel disease (IBD) are two diseases associated with FFAR2 [14]. FFAR2 has 19 associations in GWAS catalog and are related to immune and blood cells. The third gene was Major Histocompatibility Complex, Class I (HLA-K) a pseudogene with 12 associations in GWAS catalog; top trait was macrophage inflammatory protein 1b levels. The fourth gene was the Late Endosomal/Lysosomal Adaptor, MAPK And MTOR Activator 4 (LAMTOR4), a protein-coding gene associated with mTOR signaling pathway, regulation of cell size, and positive regulation of TOR signaling. LAMTOR4 has 12 transcripts and 2 GAWAS associations in GWAS catalog related to venous thromboembolism and Alzheimer disease.

#### Annotation of genes associated with DE regions between male and female (sex differences)

The set of genes associated with DE regions between male and female is listed in Supplementary Table 1. Among the list of the genes is S100A8 (S100 calcium binding protein A8), linked to diseases of immune system and an indicator of macrophage activation [27]. CD44 molecule (Indian blood group) and C-type lectin domain family 2 member B are in the list of genes, and both are linked to innate immune system. We found tetratricopeptide repeat domain 9 genes associated with DE regions, and this gene is linked to mammary gland development pathway. Interestingly, we found ring box protein-1 in the list of genes, ring box linked to invasive bladder transitional cell carcinoma and associated with a poor prognosis and tumor progression in esophageal cancer [28]. Several DE regions are annotated as lncRNA genes like X-inactive specific transcript (XIST) and Testis Expressed Transcript, Y-Linked 14 (TTTY14). We found the ZFX antisense RNA 1 (ZFX-AS1) is associated with DE TSS and enhancers between male and female.

In the Human Ageing Genomic Resources [17], the DE promoter TSS region chr6:366,786,54-366,787,97;+ overlapped all transcripts (*n*=8) from HGCN cyclin-dependent kinase inhibitor 1A (CDKN1A) gene, a member of human CD1 gene family. CDKN1A (coding for P21), a protein coding gene, is involved in several KEGG pathways (e.g., FoxO signaling pathway, p53 signaling pathway, cellular senescence, and different cancer type pathways) [29]. In response to DNA damage, CDKN1A involved in TP53 mediated inhibition of cellular proliferation [30]. Interestingly, a SNP in CDKN1A was significantly associated with longevity in a cohort from the Bologna group in central Italy [31].

#### GWAS-LD enrichment analysis of DE TSS and enhancers for frailty

GWAS-LD enrichment of analysis of the 19 DE regions (overlapped with refTSS) (non-frail vs. frail) using the GWAS-LD tool identifies GWAS traits associated with the DE regions (Fig. 2B). Top GWAS traits in (Fig. 2B) were the plantar warts, isovolumetric relaxation time, hemoglobin, and frontal fibrosing alopecia (FDR 0.0053). Other GWAS traits associated with the DE regions are major depressive disorder lifetime and urinary tract infection frequency, a common infection in old adults.

### Observed expression variations of the top differentially expressed TSS and enhancers

We looked at expression variations and annotation of the top differentially expressed TSS regions between non-frail and frail participants (Fig. 2C, D) and active enhancers regions (Fig. 2E). The TSS region chr1:207104120-207104377;+, located upstream of the Complement Component 4 Binding Protein Alpha (C4BPA) gene. A cancer cell line study [32] demonstrated that C4BPA was expressed intracellularly in cancer cells and interacts with the NF-κB family member RelA and regulates apoptosis. The same study found that C4BPA mutations are associated with improved cancer survival outcome. The second TSS region was chr2:230927722-230927775;−. This region overlapped Ensemble gene ENSG00000283164, a novel transcript of type lncRNA, antisense to G protein-coupled receptor 55 (GPR55) gene. Experimental evidence [33] and sequence similarity analysis suggested that GPR55 may be involved in hyperalgesia associated with inflammatory and neuropathic pain and may play a role in bone physiology. The third TSS region was chr11:21753419-21753559;+. This region overlapped 56 variants in Ensemble gene variant table. All variants’ consequences are intergenic variants associated with the ncRNA and uncharacterized gene LOC102723370. The fourth TSS region was chr10:133565532-133565621;−. This region overlaps the protein coding gene synaptonemal complex central element protein 1 (SYCE1). SYCE1 belongs to disease-related genes in The Human Protein Atlas [15], and diseases associated with SYCE1 include spermatogenic failure and premature ovarian failure [14]. The fifth TSS region was chr21:46605059-46605167;− overlapping protein coding gene S100 Calcium Binding Protein B (S100B). In the GeneCards database, two diseases are associated with S100B gene, syringoma, and neurofibroma. The sixth TSS region was chr22:22758650-22758750;+. This region overlaps the Immunoglobulin Lambda Variable 2-14 gene (IGLV2-14) involved in adaptive immunity [34].

We focused more on the locus OVCH1-AS1 of type long non-coding and belongs to the antisense (AS) RNA gene group and play a role in the regulation of vascular aging. AS-lncRNA mainly affects the function of endothelial cells (ECs) and vascular smooth muscle cells (VSMCs.) [35]. The barplot in Fig. 2D shows significant difference in the expression of OVCH1-AS1 between frail and non-frail participants. In the GWAS catalog, OVCH1-AS1 overlaps 10 variant and risk alleles. Alzheimer disease, gut microbiota, and alkaline phosphatase are among the reported traits associated with OVCH1-AS.

CAGE also enabled testing whether a gene shows differential TSS usage or not for genes with multiple TSSs. Supplementary Fig. 4 shows alternative TSS usage within genes, most of the genes used only a single TSS, and only a minority of genes used 2 or more TSSs (up to 7 TSSs).

### Analysis of the expression of age-related genes between non-frail and frail participants

**Fig. 3:**
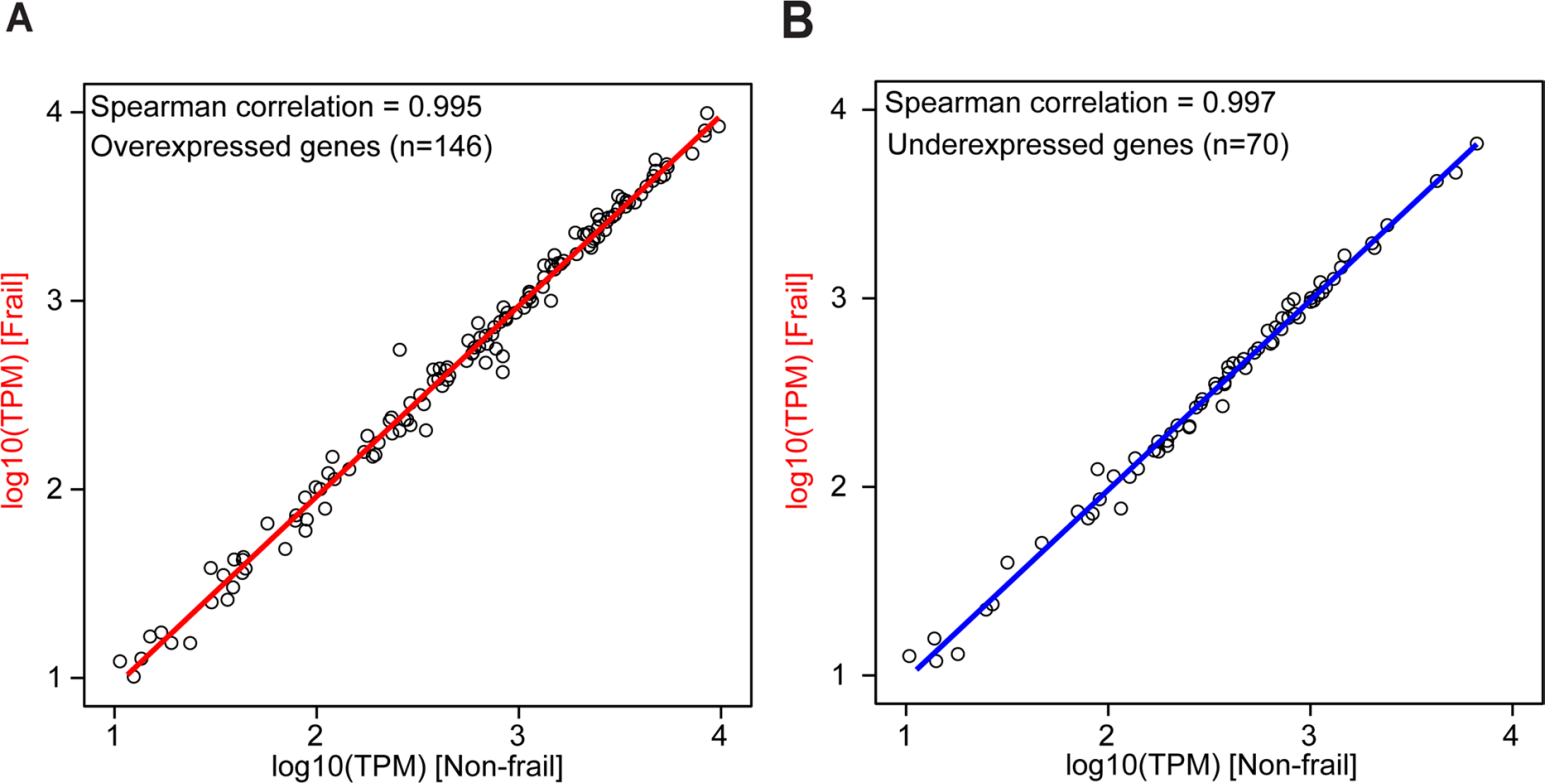
Correlation analysis of the expression of age-related gene between non-frail and frail. Correlation analysis of the age-related gene expression between non-frail and frail subjects. **A** Over-expressed genes and **B** under-expressed genes. Source of the genes [36]

We investigated the expression of the previously described age-related genes from a meta-analysis of the multiple age-related gene expression profiles [36]. We tested the correlation of the expression of the age-related genes between non-frail and frail participants; the correlation analysis shows strong Spearman’s correlation for both over-expressed (Fig. 3A) and under-expressed genes (Fig. 3B).

### Weighted gene co-expression network analysis

**Fig. 4:**
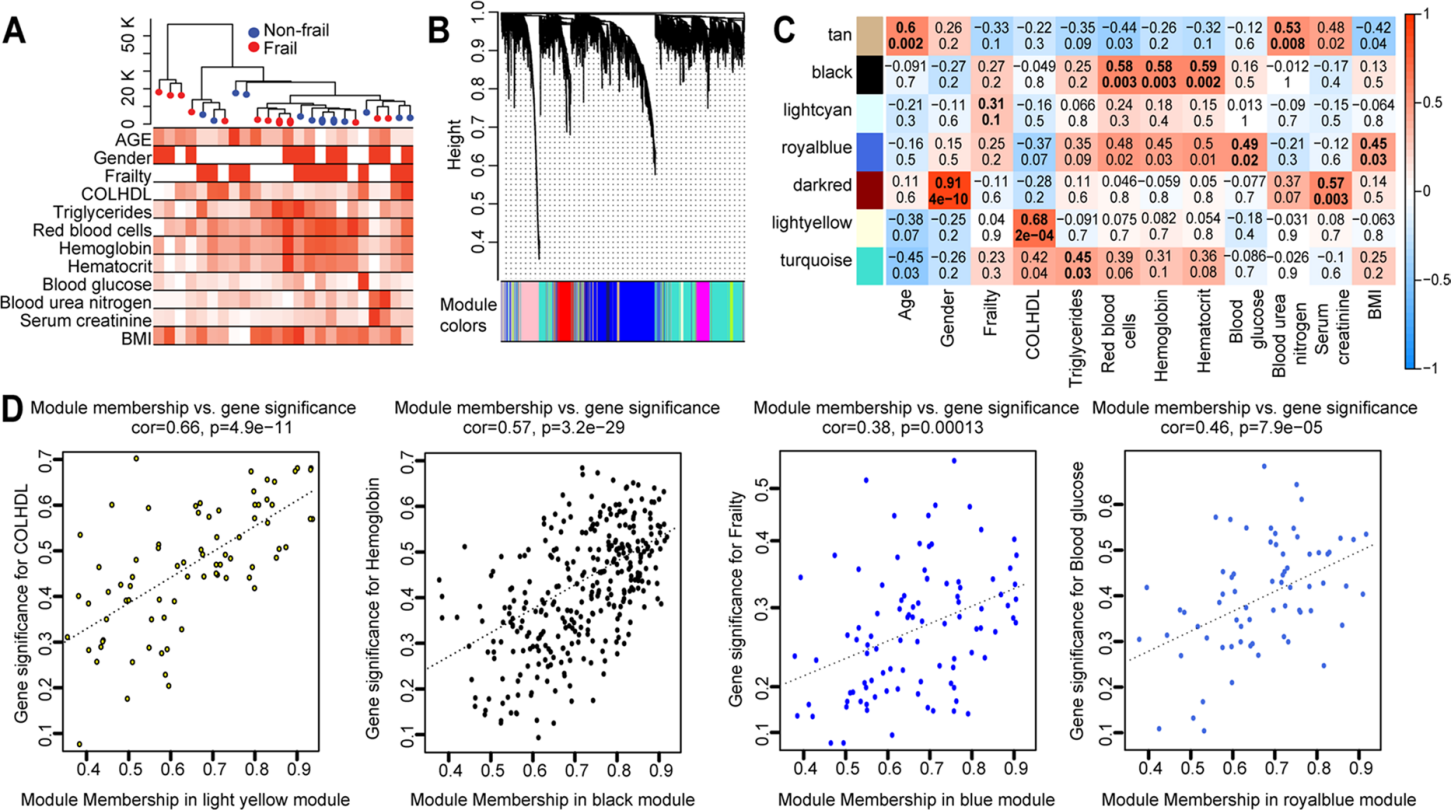
Weighted correlation network analysis. **A** Clustering of sample expression profiles. The dendrogram based on sample Euclidean distance (top) and heatmap of the basic demographic information, frailty data, clinical variable, and BMI. In the heatmap, white means a low value and red means a high value for continuous variables (age, COLHDL, triglycerides, red blood cells, hemoglobin, hematocrit, blood glucose, blood urea nitrogen, serum creatinine, and BMI). For gender, white means female and red means male. For frailty, white means frail and red means non-frail. **B** Genes clustering dendrogram with the assigned module colors (22 modules). Genes dissimilarity based on topological overlap were computed by blockwiseModules function using automatic network construction and module detection. **C** Association between module eigengene and demographic and clinical data. Rows correspond to a module eigengene and column to demographic and clinical data. Cells are color-coded by correlation strength. Cell contains the significance trait/module correlation (top number) and *P*-value (bottom number). Cells with the highest correlation value are set in bold. **D** Scatterplot of gene significance vs. module membership. Cholesterol HDL (COLHDL) (left) in the light-yellow module and hemoglobin (right) in the back module. Both scatterplots show high significant correlation between the gene significance and light yellow and black module membership

The weighted gene co-expression network analysis (WGCNA) enabled the integration of the expression matrix for each sample and their corresponding demographic and clinical laboratory data. The gene expression matrix for all participants was used as input for the WGCNA, and the samples were clustered based on the expression matrix. This analysis did not show any evident outliers in the data. The dendrogram in Fig. 4A was annotated by the frailty variable (non-frail and frail)

#### Gene network with 22 modules identified

The gene clustering dendrogram with the assigned module colors is shown in Fig. 4B. For the 22 modules, the minimum module significance (the average absolute gene significance measure for all genes in a given module [8]) is 0.1204, mean is 0.1889, and maximum module significance is 0.3959. Furthermore, we investigated the module-trait associations and found significant associations shown by the coefficient correlation in (Fig. 4C and Table 2). As an example, in Fig. 4C and Table 2, the tan color module is significantly associated with age (*r* = 0.6), while the black color module is significantly associated with red blood cells, hemoglobin, and hematocrit (*r*= 0.58). To find the association of individual gene in the network and particular traits, WGCNA uses two measures to identify a set of genes with high significance with clinical traits as well the high module member genes in specific module(s), the gene significance (GS), and module membership (MM) (see “Methods”). Fig. 4D shows the scatterplots of GS vs. MM for two modules; the light-yellow module left panel of Fig. 4D for the COLHDL trait shows a strong and highly significant correlation (*r*=0.66; *P*-value 4.9e−11). The black module right panel in Fig. 4D shows also strong and significant correlation (*r*=0.57; *P*-value 3.2e−29). Furthermore, blue module and royal blue module in Fig. 4D showed the high correlation and strong *P*-value for frailty and blood glucose, respectively.

**Table 2:** DAVID bioinformatics tools functional annotation results for selected WGCNA modules. Correlational coefficient values ≥ 0.5 are set in bold. FDR values for enrichment terms are set in bold for FDR < 0.05

Trait	Module	Correlation coefficient	Enrichment term	FDR
Age	Tan	**0.6**	Identical protein binding	0.06
Gender	Dark red	**0.91**	Dioxygenase	ns
Frailty	Light cyan	0.31	Neutrophil extracellular trap formation	**< 0.0001**
HDL	Light yellow	**0.68**	Human papillomavirus infection	ns
Triglycerides	Turquoise	0.45	DNA damage	**< 0.0001**
Red blood cells	Black	**0.58**	Lysosome	**0.004**
Hemoglobin	Black	**0.58**	Lysosome	**0.004**
Hematocrit	Black	**0.59**	Lysosome	**0.004**
Blood glucose	Royal blue	**0.5**	Autophagosome	ns
Blood urea nitrogen	Tan	**0.53**	Identical protein binding	0.06
Serum creatinine	Dark red	**0.57**	Dioxygenase	ns
BMI	Royal blue	0.45	Autophagosome	ns

#### Functional annotation of the gene modules with strong significance trait/module correlation

Out of the identified 22 gene modules from WGCNA, we selected 7 modules with significant trait/module correlation (Fig. 4C) and performed functional annotation of their module member genes. The results of DAVID [16] functional annotation of the genes in 7 gene modules are shown in Table 2. The gene module light cyan was associated with the frailty (non-frail and frail) which was enriched for the KEGG pathway neutrophil extracellular trap (NET formation (FDR < 0.0001). NETs are a DNA scaffold formed as a defense mechanism by neutrophil. NETs contribute to immobilization and neutralization of different microorganisms [37]. NETs play important detrimental and beneficial roles in inflammation, autoimmunity, and other pathophysiological conditions [38].

#### Distinct gene module identified by WGCNA

**Fig. 5:**
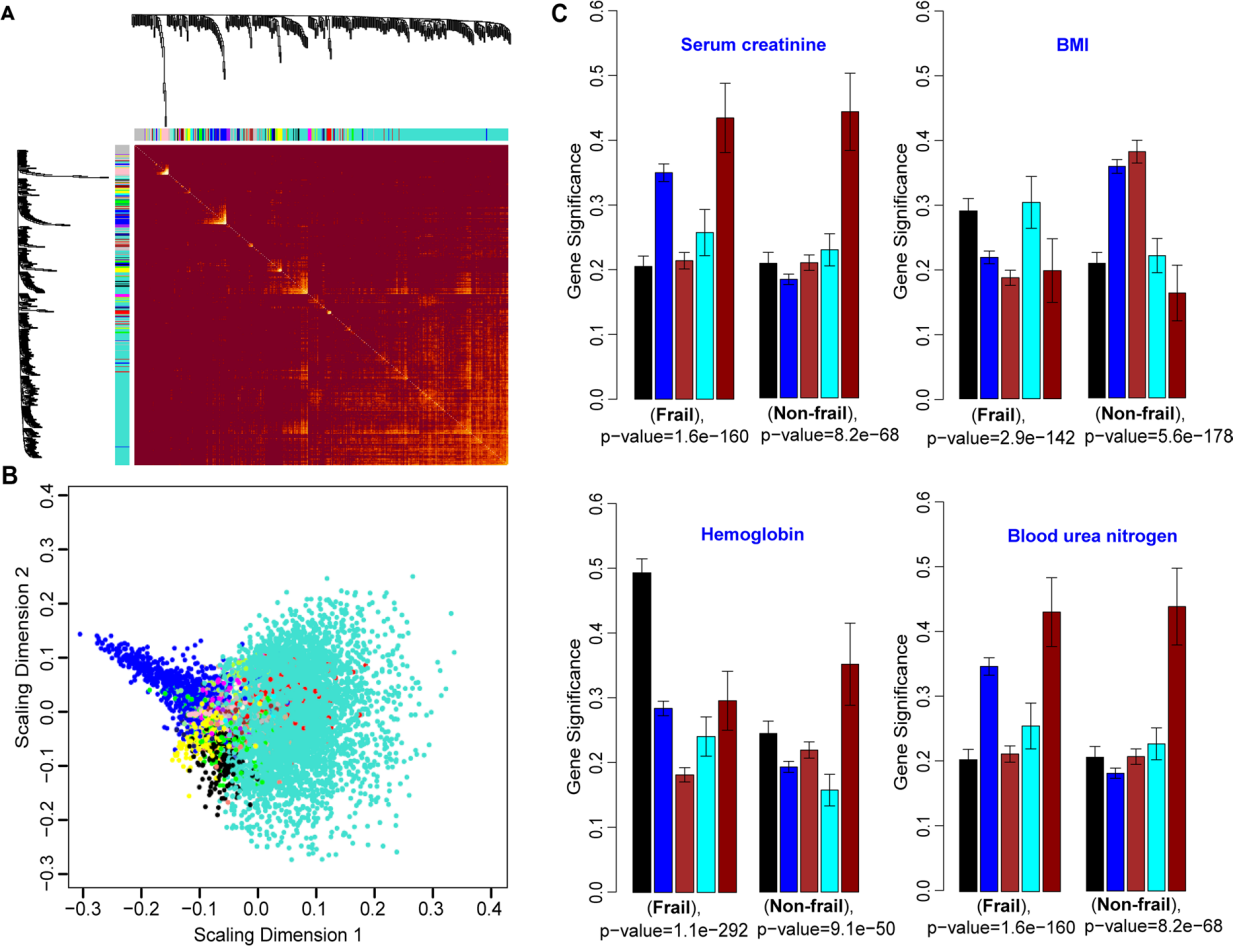
Topological overlap matrix (TOM) analysis. **A** Heatmap of the topological overlap matrix on the top and left side gene dendrogram tree and module assignment (colored). In the heatmap, light color represents low overlap genes and darker red color represents higher overlap between genes. Each row and column in the heatmap correspond to a single genes. **B** Multi-dimensional scaling plot of module members. **C** Barplot of module significance (black, blue, cyan, red, and dark red (five modules)). The module significance is defined as the mean gene significance across all genes in specific module. For each clinical test (serum creatinine, hemoglobin, BMI, and blood urea nitrogen), two barplots are shown for frail (left) and non-frail (right)

The constructed gene network obtained by the WGCNA is visualized in Fig. 5A. The heatmap in Fig. 5A shows the topological overlap matrix (TOM) of all genes. In the heatmap, the 22 modules from the gene network are shown diagonally with very low overlap between them (indicated by blocks of light color). The heatmap of TOM was visualized together with the gene module dendrogram on top and left side of the heatmap and the module colors at the top and left edge of the heatmap. In addition to the TOM heatmap of all gene network, we identified meta-modules (a group of correlated eigengenes) per each trait. The eigengene dendrogram and the eigengene adjacency heatmap in Supplementary Fig. 5 visualize the relationship among the modules and the clinical traits (one dendrogram and heatmap per each trait).

To find the distinct module from the 22 gene modules, we used the multi-dimensional scaling plot (Fig. 5B) which revelated that the blue module is the most distinct module with 1045 genes and module significance (the average absolute gene significance measure for all genes in a given module [8]) of 0.171. We analyzed the module significance for five modules and four clinical traits related to frailty status (serum creatinine, hemoglobin, BMI, and blood urea nitrogen) (Fig. 5C). Each panel in Fig. 5C shows the module significance for the frail and non-frail subjects and p-value. The association between the dark red module gene and serum creatinine was the highest in both frail and non-frail participants, and similarly, blue module shows highly significant association, although *P*-values are somewhat different between frail and non-frail. For the BMI, the blue module has higher gene significance in non-frail compared to frail. Similar consideration applies to blue module correlations within hemoglobin and blood urea nitrogen (Fig. 5C).

#### Immune system related pathways and GO terms are enriched in the identified gene modules

**Fig. 6:**
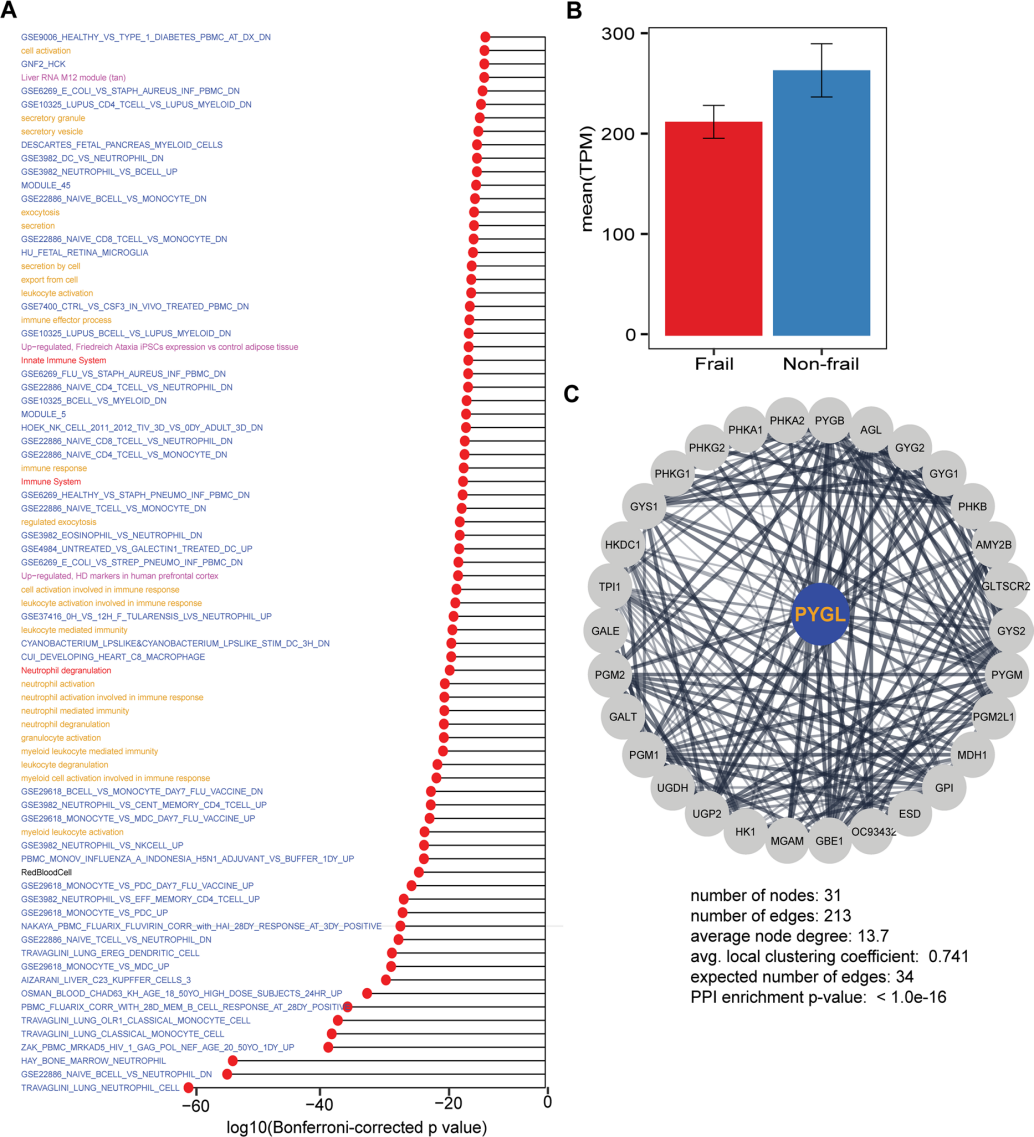
Gene set enrichment analysis of blue module and hub genes identification. **A** Gene set enrichment analysis of blue module for the top enriched term from Reactome pathway database (red labels), Molecular Signatures Database v7.4 (blue labels), WGCNA internal collection JAM (black label), HD Molecular Signatures–HDSigDB (magenta labels), and Gene ontology BP|CC (yellow labels). **B** Expression profile of the 20 hub genes in the blue module (non-frail vs. frail); data in the bar chart are represented as expression ± SEM. **C** Protein-protein-interaction network (PPI) for the PYGL (Glycogen Phosphorylase L) protein coding gene and blue module hub gene

To understand the biological meaning and relevance of the identified gene modules from WGCNA, we performed gene set enrichment analysis (GSEA) of the top enriched genes in the blue module using the anRichment R package, which relays on enrichment annotations from several databases (Reactome pathway database, Molecular Signatures Database v7.4, WGCNA internal collection JAM, HD Molecular Signatures–HDSigDB, and Gene ontology for biological process and cellular compartment). The list of all enrichment terms for each module with their *P*-value, FDR, and *Bonferroni’s correction* is shown in Supplementary Table 2. The visualization of enrichment terms for the blue module (distinct module) is shown in Fig. 6A. Most of the enriched terms are related to immune system, e.g., the Reactome terms neutrophil degranulation, immune system, and innate immune system and also the enriched GO BB terms myeloid leukocyte activation, neutrophil activation, leukocyte degranulation, myeloid cell activation involved in immune response, cell activation involved in immune response, and others.

#### Protein-protein interaction (PPI) network analysis of the hub genes in blue module

We export the blue module genes to Cytoscape and identified the hub nodes in the network (genes) (“Methods”). Fig. 6B shows the expression profile of the 20 hub genes in the blue module, which indicated that non-frail subjects have higher mean expression of the hub genes compared to the frail subjects. Finally, we looked at the PPI of the top hub gene Glycogen Phosphorylase L (PYGL) as shown in (Fig. 6C) with PPI enrichment *P*-value < 1.0e−16 a PYGL code for protein that is involved in cAMP-dependent activation of PKA [14].

### Members of ETS (erythroblast transformation-specific) family TF enrichment

**Fig. 7:**
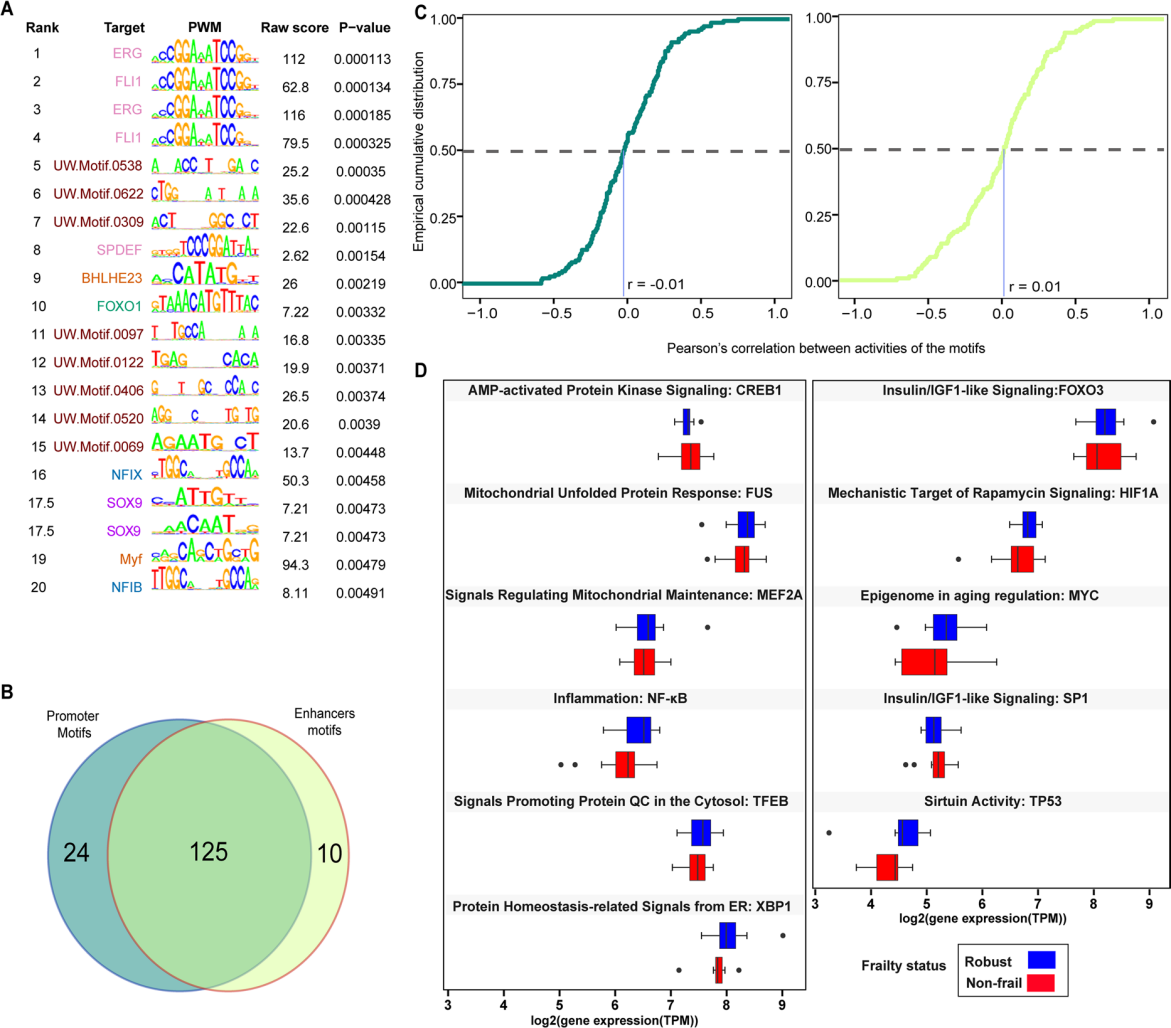
Transcription factors biding site prediction and motifs activity analysis. **A** 20 predicted transcription factor binding sites (TFBS). Each TFBS was ranked based on the *P*-value and the target colored by the TF family. The first column is the rank, and the second shows the target name, which is either a gene name, an isoform name, or a dimer name. The next column in the plot is the PWM logo, following the motif ID. This ID comes from the MotifDb package. The next-to-last column is the raw affinity score, and the last column is the *P*-value of motif enrichment. **B** Venn diagram of the promoter vs. enhancer transcription factors. **C** Cumulative distribution (cd) of Pearson’s correlation r for the promoters (left) and enhancers (right) between non-frail and frail. The median value of persons’ correlation *r* on the horizontal axis of each graph. **D** log_2_ expression of the selected transcription factors (TF) involved in age-related pathways between non-frail and frail subjects

We looked at the enrichment of DNA-binding motifs for the differentially expressed TSSs and active enhancers (see “Methods”). Based on motif enrichment scores, the top 20 ranked motifs are shown in Fig. 7A. Among the top ranked motifs, we identified 3 gene members of the ETS transcription factor family [39]. Those are the ETS-related gene subfamily (ERG and FLI1) and prostate-derived Ets transcription factor subfamily (SPDEF) which play key roles as differentiation regulators and tumorigenesis in endocrine organs and target tissues [39]. ERG (ETS Transcription Factor ERG), FLI1 (Fli-1 proto-oncogene), and SPDEF (SAM Pointed Domain Containing Ets Transcription Factor) are all protein coding genes that play a role as a DNA-binding transcription activator. Also, we found that two members of the basic helix-loop-helix proteins (BHLH) were enriched, the Basic Helix-Loop-Helix Family Member E23 (BHLHE23) and myogenic factor 5 (myf) which is a transcriptional repressor. Among the top 20 ranked motifs, we found the Forkhead box protein O1 (FOXO1) a member of the forkhead family of transcription factors known as longevity associated genes by alterations in the insulin/IGF pathway. The enriched FOXO1 TF is a prolog of FOXO3, a gene that carries polymorphisms that have been strongly associated with longevity in genome-wide association studies [14]. Among the top ranked TF was the nuclear factor I X (NFIX) and nuclear factor I B (NFIB) both belonging to nuclear factor I family (NFI). Both NFIX and NFIB play a key role in development and cancer [40]. The SRY-Box Transcription Factor 9 (SOX9) a member of SRY-box transcription factors (SOX) was enriched as well. SOX9 is a protein coding gene and transcription activator which plays a key role in chondrocytes differentiation and skeletal development [41].

### Motif activity analysis and aging-regulatory signaling pathway analysis

#### Motif activity of promoter and enhancer TF between non-frail and frail

To investigate the motif activity of promoter and enhancer TF between non-frail and frail, we first estimated TF using the identified TSS and enhancers (“Methods”). Overall, we predicted 159 TFs. 125 of them are common for both promoters and enhancers, 24 are promoter specific TF, and 10 are enhancer specific TF as shown in Venn diagram (Fig. 7B).

We calculated the motif activity for promoter and enhancer (“Methods”). The raw calculated motif activity is shown in Supplementary Tables 3 and 4. The motif activity values for promoters and enhancers are plotted with quantile-quantile (Q-Q) plot in Supplementary Fig. 6. We started by estimating Pearson’s pairwise correlation between non-frail and frail for 149 promoter activity and next we repeat the same procedure and tested Pearson’s correlation between non-frail and frail for 135 enhancer activities (Supplementary Table 5). To test the difference in Pearson’s pairwise correlations between non-frail and frail (promoter and enhancer TF), we computed and plotted the empirical cumulative distribution (CDF) (Fig. 7C). The median value of persons’ correlation *r* on the horizontal axis of each graph in (Fig. 7C) indicates that more than half of the promoter activity of frail subjects negatively correlated (inverse relationship) with the promoter activity of non-frail subjects (mean *r* = −0.01). Interestingly, among the top ranked promoters, we found that the Engrailed Homeobox 1 and 2 (EN1,2). EN1,2 a protein coding gene has been implicated in pattern formation during development of the central nervous system and among its enriched pathway dopaminergic neurogenesis [42]. Likewise, for the half of the enhancers, the activity in frail subjects is negatively correlated with the enhancer activity of non-frail subjects (mean *r* = 0.01) of all enhancers.

#### CAGE expression of the major aging-regulatory signaling pathways

After defining the set of TFs, we looked at the CAGE expression of the major aging-regulatory signaling pathways and their downstream transcription factors which was curated from [43]. The Box-and-whisker plot in Fig.7D shows the variation in CAGE expression for each category of major aging-regulatory signaling pathways between non-frail and frail.

## Discussion

The aim of this study was to integrate information from whole-blood gene expression and clinical data (traits) to shed light on the biological mechanisms that distinguish non-frail versus frail individuals in a subset of participants from the InCHIANTI study. The analysis of the gene expression from CAGE shows that TSS annotated promoters are highly expressed compared to unannotated TSSs (novel TSSs) and enhancer expression is lower than that of TSSs as reported before [44–46].

Performing differential expression analysis, between non-frail and frail and male and female participants, we identified a set of DE TSSs and active enhancer region with *Padj* < 0.05. We performed genomic and functional annotation of the DE regions. Several DE TSS are annotated as protein coding, lncRNA, or antisense RNA. The GWAS-LD enrichment analysis of the DE regions of frailty revealed a set GWAS reported traits associated with the DE regions. Several cardiovascular, psychiatric disorders, and infection related traits are found to be significantly associated with DE regions.

We looked closely at the expression of the top differentially expressed promoters and enhancers between non-frail and frail participants. Complement component 4 binding protein alpha (C4BPA) was among the top differentially expressed promoters and lowly expressed in frail subjects (*P* < 0.01) (Fig. 2C). The protein encoded by this gene plays key roles in immunity and innate immunity and has been found to be enriched in immune cell (neutrophil) in The Human Protein Atlas [15] and highly expressed in the liver, lung, and whole-blood tissue in the Genotype-Tissue Expression (GTEx) [47]. Another gene highly expressed in frail subjects was G protein-coupled receptor 55 (GPR55) which play key role in several physiological and pathological processes by activating a variety of signal transduction pathways [15]. We also found the gene OVCH1-AS1 among the differentially expressed genes. OVCH1-AS1 is a lncRNA and belongs to an antisense RNA gene group. Aging studies showed that several lncRNA genes play a role during aging by mediating cellular senescence in difference phases of cell cycle by modulating senescence-associated pathways [48]. As special lncRNA, the antisense RNA plays a role in the regulation of vascular aging and mainly affects the function of endothelial cells (ECs) and vascular smooth muscle cells (VSMCs) [35]. The overexpression of OVCH1-AS1 in non-frail participants is compared to the low level of the expression in frail participants, indicating that this gene could be a discriminant marker for frailty and could be explored as a target in future investigations. The correlation of the obtained gene expression of the list of published age-related genes between non-frail and frail subjects shows strong Spearman’s correlation for both over-expressed and under-expressed genes. These findings suggest that the differential expression of specific gene with both aging and frailty is modulated by similar differential expression of TSS and enhancers.

WGCNA analysis identifies 22 modules related to 12 clinical traits. Among these modules, we identified the blue module as a distinct module. The gene significance of the blue module discriminates several of the clinical traits that have been associated with frailty (BMI, blood urea nitrogen, serum creatinine, and hemoglobin). We considered the blue module for the gene set enrichment analysis to understand the biological relevance of the set of gene in the module. Not surprisingly, we found that most of the enriched terms are related to immune system and innate immune system. The enriched GO terms are myeloid leukocyte activation, neutrophil activation, leukocyte degranulation, myeloid cell activation involved in immune response, cell activation involved in immune response, etc. We also took the set of genes in the blue module and performed network analysis to find the top hub genes. The highest top hub gene was Glycogen Phosphorylase L (PYGL) which is involved in carbohydrate metabolism and glycogen metabolism. PYGL is related to activation of cAMP-dependent PKA pathway [14].

DAVID functional enrichment analysis of the 7 gene modules with strong trait/module correlation shows that the light cyan module (frailty) enriched for the neutrophil extracellular trap formation, a mechanism used to by neutrophil to immobilize microorganisms. Neutrophil extracellular trap (NET) formation plays important roles in autoimmune, inflammatory, and different pathophysiological conditions [38]. The enrichment of NETs formation pathway is a drug target for several drugs, such as Mitiperstat that is used treat heart failure and Imiquimod an antiviral and immunomodulator.

The 20 top ranked target genes resulting from transcription factor binding site (TFBS) analysis included ERG, FLI1, and SPDEF genes which belong to erythroblast transformation-specific (ETS) family of TF. This is consistent with the well-known association between both aging and frailty with anemia. Members of ETS family play a key role as regulators of cell proliferation, differentiation, angiogenesis, inflammation, and apoptosis, and they are cancer-related genes and proto-oncogene [15, 42]. In addition to ERG and FLI1, we identified Forkhead box protein O1 (FOXO1) member of the forkhead box genes associated with longevity [49] and defined as the main target of insulin signaling and a key regulator of metabolic homeostasis in response to oxidative stress [50]. We identified in the list of target gene SOX9, a TF which plays a key role in skeletal development in chondrocyte differentiation [41].

The motif activity analysis shows significant differences in motif activity between frail and non-frail. Finally, we analyzed the expression of the TF of the aging-regulatory signaling pathways which also shows differences in the expression profile between frail and non-frail.

The strengths of our study include the integration of gene expression profiling and clinical traits using network approach and statistical methods. This is the first study in the literature that uses a complex bioinformatic pipeline to characterize CAGE expression profile of old adults in a well-characterized cohort and employ different statistical methods to integrate expression data with clinical traits.

Our findings were obtained using data and samples from participants of the InCHIANTI study, a well-characterized, high-quality cohort. Our study also has limitations. For example, while we were able to identify a set of differentially expressed TSSs and active enhancers, we could not validate these findings in an independent population. In addition, because of the small sample size (*n*=24), our findings should be interpreted with cautions and should be validated in future studies.

In conclusion, the CAGE profiles enabled us to identify several lnRNAs and antisense RNA differentially expressed between non-frail and frail participants. WGCNA analysis identifies PYGL gene as a hub gene in the identified gene modules. Furthermore, we found novel association between motif activities and frailty status. Our study will help to understand the regulation of longevity-related genes. The set of promoters and active enhancers identified in this study could be explored in different ways and can be re-used for further studies.

## Statistical analysis

R statistical package version 4.1.2 (2021-11-01) was used for all analyses. Data are represented in box and whisker plot, boxplots, and bar plots (represented as expression ± SEM) as specified in the figure legends. For testing differentially expressed TSS and enhancers, *F*-statistic, the associated *P*-value, and the *adj. P*-value were corrected using the Benjamini-Hochberg correction.

### Supplementary information

ESM 1 (XLSX 39 kb)

ESM 2 (XLSX 108 kb)

ESM 3 (XLSX 100 kb)

ESM 4 (XLSX 153 kb)

ESM 5 (XLSX 11 kb)

ESM 6 (JPG 609 kb)

ESM 7 (JPG 2058 kb)

ESM 8 (JPG 207 kb)

ESM 9 (JPG 587 kb)

ESM 10 (JPG 548 kb)

ESM 11 (JPG 2674 kb)

ESM 12 (DOCX 56.4 kb)

ESM 13 (XLSX 227 kb)

## Data Availability

The following datasets are available as R data object (.rds) for download from the public website https://dmsgrdm.riken.jp:5000/4jt3q/: - Identified TSSs and active enhancers - List of DE regions between non-frail and frail participants - List of DE regions between male and female participants All raw data were deposited to a controlled-access database, and the detail of the access and the name of the database were provided in https://dmsgrdm.riken.jp:5000/4jt3q/.

## References

[1] Fried LP (2001). Frailty in older adults: evidence for a phenotype. J Gerontol A Biol Sci Med Sci..

[2] Lippi G (2015). Laboratory biomarkers and frailty: presentation of the FRAILOMIC initiative. Clin Chem Lab Med..

[3] Debès C (2023). Ageing-associated changes in transcriptional elongation influence longevity. Nature.

[4] Murata M (2014). Detecting expressed genes using CAGE. Methods Mol Biol.

[5] Takahashi H (2021). Low quantity single strand CAGE (LQ-ssCAGE) maps regulatory enhancers and promoters. Methods Mol Biol.

[6] Shiraki T (2003). Cap analysis gene expression for high-throughput analysis of transcriptional starting point and identification of promoter usage. Proc Natl Acad Sci USA.

[7] Love MI (2014). Moderated estimation of fold change and dispersion for RNA-seq data with DESeq2. Genome Biol.

[8] Langfelder P, Horvath S (2008). WGCNA: an R package for weighted correlation network analysis. BMC Bioinform.

[9] Ferrucci L (2000). Subsystems contributing to the decline in ability to walk: bridging the gap between epidemiology and geriatric practice in the InCHIANTI study. J Am Geriatr Soc.

[10] Pedone C (2023). Predicting risk of declining functional ability in community-dwelling older people. Arch Gerontol Geriatr.

[11] Abugessaisa I (2017). FANTOM5 CAGE profiles of human and mouse reprocessed for GRCh38 and GRCm38 genome assemblies. Sci Data.

[12] Abugessaisa I (2019). refTSS: a reference data set for human and mouse transcription start sites. J Mol Biol.

[13] Quinlan AR, Hall IM (2010). BEDTools: a flexible suite of utilities for comparing genomic features. Bioinformatics.

[14] Safran M, Abugessaisa I, Kasukawa T (2021). The GeneCards Suite. Practical Guide to Life Science Databases.

[15] Uhlén M, et al. Proteomics. Tissue-based map of the human proteome. Science. 2015;347(6220) 126041910.1126/science.1260419 10.1126/science.1260419 25613900

[16] Dennis G (2003). DAVID: Database for Annotation, Visualization, and Integrated Discovery. Genome Biol.

[17] Tacutu R (2018). Human ageing genomic resources: new and updated databases. Nucleic Acids Res.

[18] Machiela MJ, Chanock SJ (2015). LDlink: a web-based application for exploring population-specific haplotype structure and linking correlated alleles of possible functional variants. Bioinformatics.

[19] Sollis E (2023). The NHGRI-EBI GWAS catalog: knowledgebase and deposition resource. Nucleic Acids Res.

[20] Shannon P (2003). Cytoscape: a software environment for integrated models of biomolecular interaction networks. Genome Res.

[21] Suzuki H (2009). The transcriptional network that controls growth arrest and differentiation in a human myeloid leukemia cell line. Nat Genet.

[22] Rosin P, Rammler E (1933) The law governing the fineness of powdered coal. J Inst Fuel 7:29–36 and discussion, pp 109–122

[23] Carninci P (2006). Genome-wide analysis of mammalian promoter architecture and evolution. Nat Genet.

[24] Abugessaisa I, et al. FANTOM5 transcriptome catalog of cellular states based on Semantic MediaWiki. Database (Oxford). 2016;2016 10.1093/database/baw105. 10.1093/database/baw105 PMC4940433 27402679

[25] Xu JZ (2018). Antisense RNA: the new favorite in genetic research. J Zhejiang Univ Sci B.

[26] Razazan A (2021). Activation of microbiota sensing - free fatty acid receptor 2 signaling ameliorates amyloid-β induced neurotoxicity by modulating proteolysis-senescence axis. Front Aging Neurosci.

[27] Wang S (2018). S100A8/A9 in inflammation. Front Immunol.

[28] Kunishige T (2020). Ring box protein-1 is associated with a poor prognosis and tumor progression in esophageal cancer. Oncol Lett.

[29] Kanehisa M (2021). KEGG: integrating viruses and cellular organisms. Nucleic Acids Res.

[30] Zahn-Zabal M (2020). The neXtProt knowledgebase in 2020: data, tools and usability improvements. Nucleic Acids Res.

[31] Gravina S (2009). Identification of single nucleotide polymorphisms in the p21 (CDKN1A) gene and correlations with longevity in the Italian population. Aging (Albany NY).

[32] Olcina MM (2020). Intracellular C4BPA levels regulate NF-κB-dependent apoptosis. iScience..

[33] Whyte LS (2009). The putative cannabinoid receptor GPR55 affects osteoclast function in vitro and bone mass in vivo. Proc Natl Acad Sci USA.

[34] UniProt Consortium T (2018). UniProt: the universal protein knowledgebase. Nucleic Acids Res.

[35] Cui XY (2021). Roles and functions of antisense lncRNA in vascular aging. Ageing Res Rev.

[36] de Magalhães JP (2009). Meta-analysis of age-related gene expression profiles identifies common signatures of aging. Bioinformatics.

[37] Branzk N (2014). Neutrophils sense microbe size and selectively release neutrophil extracellular traps in response to large pathogens. Nat Immunol.

[38] Boeltz S (2019). To NET or not to NET:current opinions and state of the science regarding the formation of neutrophil extracellular traps. Cell Death Differ.

[39] Gutierrez-Hartmann A (2007). ETS transcription factors in endocrine systems. Trends Endocrinol Metab.

[40] Chen KS (2017). The convergent roles of the nuclear factor I transcription factors in development and cancer. Cancer Lett.

[41] Matsushita M (2013). A novel SOX9 H169Q mutation in a family with overlapping phenotype of mild campomelic dysplasia and small patella syndrome. Am J Med Genet A.

[42] Maglott D (2011). Entrez gene: gene-centered information at NCBI. Nucleic Acids Res.

[43] Zhou X (2018). Regulation of age-related decline by transcription factors and their crosstalk with the epigenome. Curr Genomics.

[44] Arner E (2015). Transcribed enhancers lead waves of coordinated transcription in transitioning mammalian cells. Science.

[45] Andersson R (2014). An atlas of active enhancers across human cell types and tissues. Nature.

[46] Forrest AR (2014). A promoter-level mammalian expression atlas. Nature.

[47] Consortium G (2013). The Genotype-Tissue Expression (GTEx) project. Nat Genet.

[48] He J (2018). Role of lncRNAs in aging and age-related diseases. Aging Med (Milton).

[49] Greer EL, Brunet A (2005). FOXO transcription factors at the interface between longevity and tumor suppression. Oncogene.

[50] Valis K (2011). Hippo/Mst1 stimulates transcription of the proapoptotic mediator NOXA in a FoxO1-dependent manner. Cancer Res.

