# Supplementary figures and images for: OVCH1 Antisense RNA 1 is differentially expressed between non-frail and frail old adults
Source: GeroScience. 2023 Oct 11;46(2):2063–81. doi: 10.1007/s11357-023-00961-9 (PMC10828349; doi:10.1007/s11357-023-00961-9)

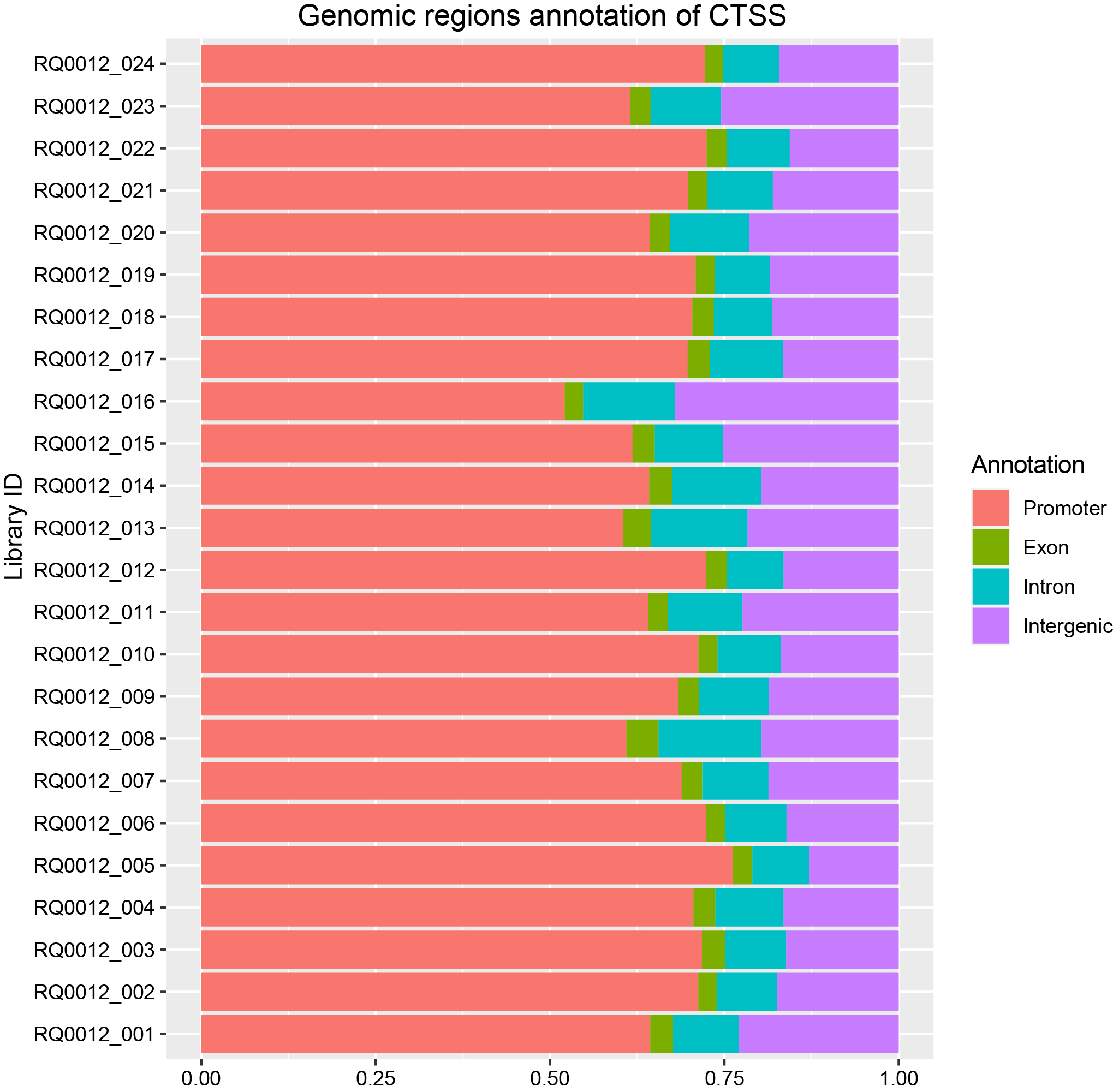

Supplement: Supplementary file 6 — (JPG 609 kb) [file 11357_2023_961_MOESM6_ESM.jpg]

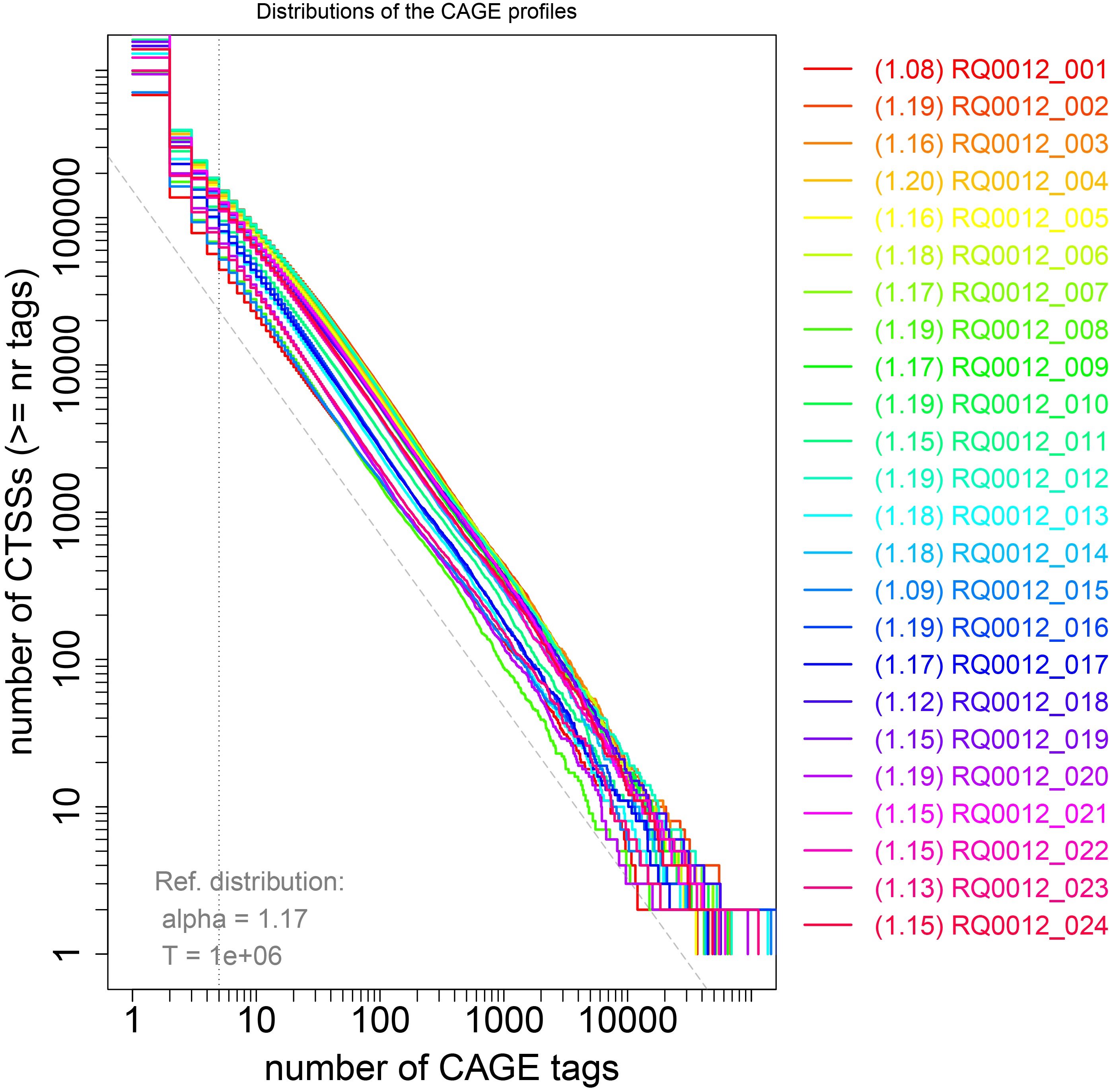

Supplement: Supplementary file 7 — (JPG 2058 kb) [file 11357_2023_961_MOESM7_ESM.jpg]

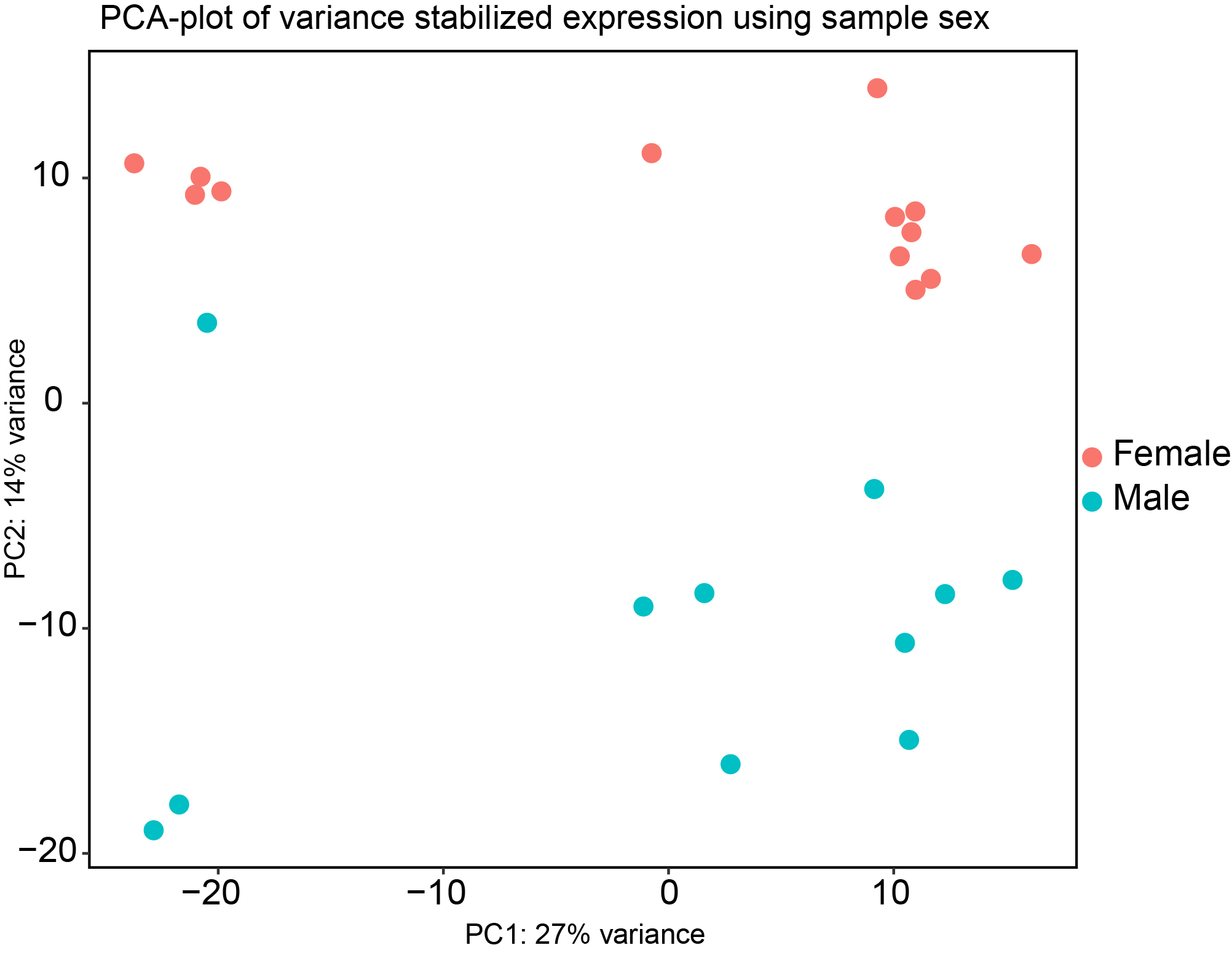

Supplement: Supplementary file 8 — (JPG 207 kb) [file 11357_2023_961_MOESM8_ESM.jpg]

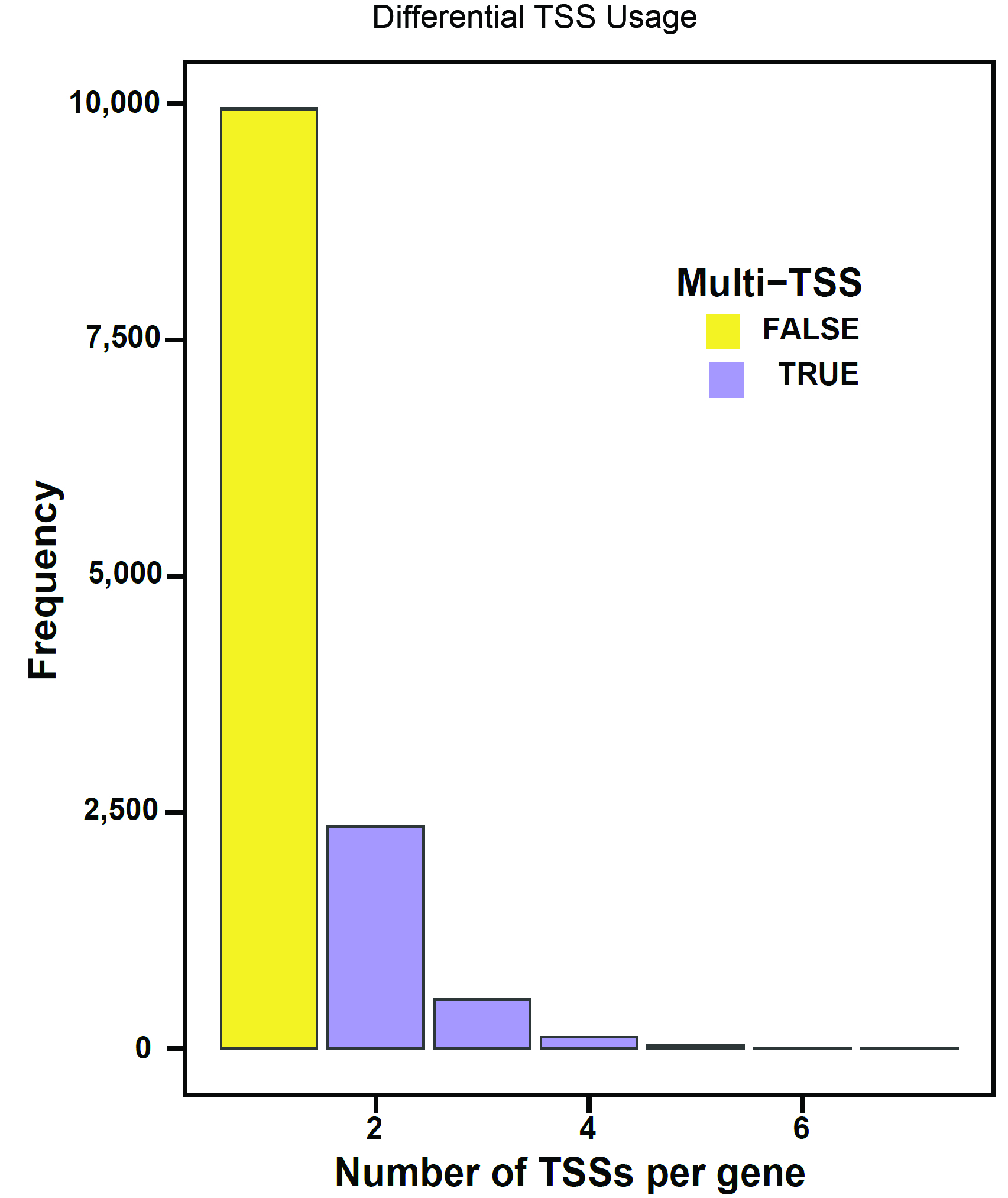

Supplement: Supplementary file 9 — (JPG 587 kb) [file 11357_2023_961_MOESM9_ESM.jpg]

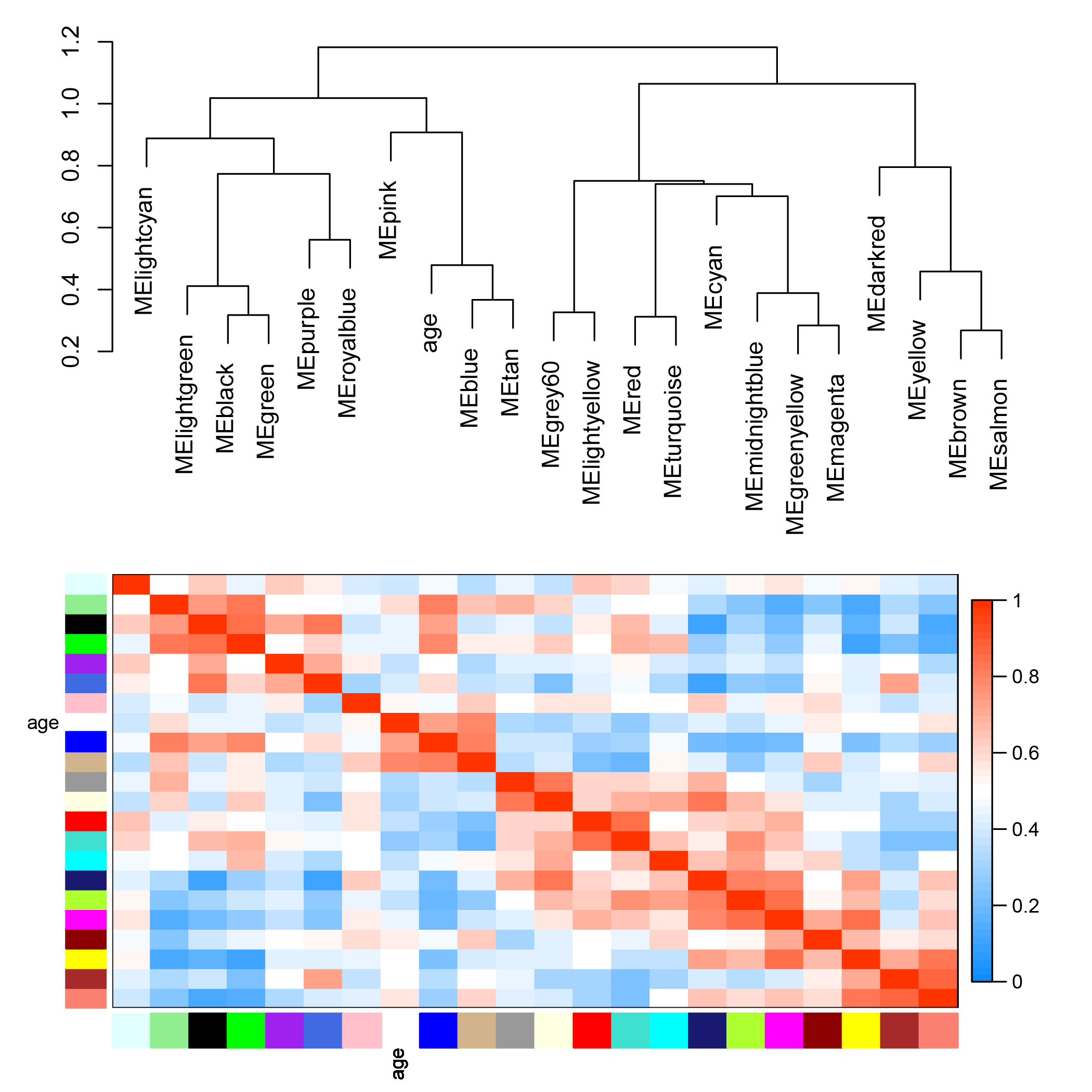

Supplement: Supplementary file 10 — (JPG 548 kb) [file 11357_2023_961_MOESM10_ESM.jpg]

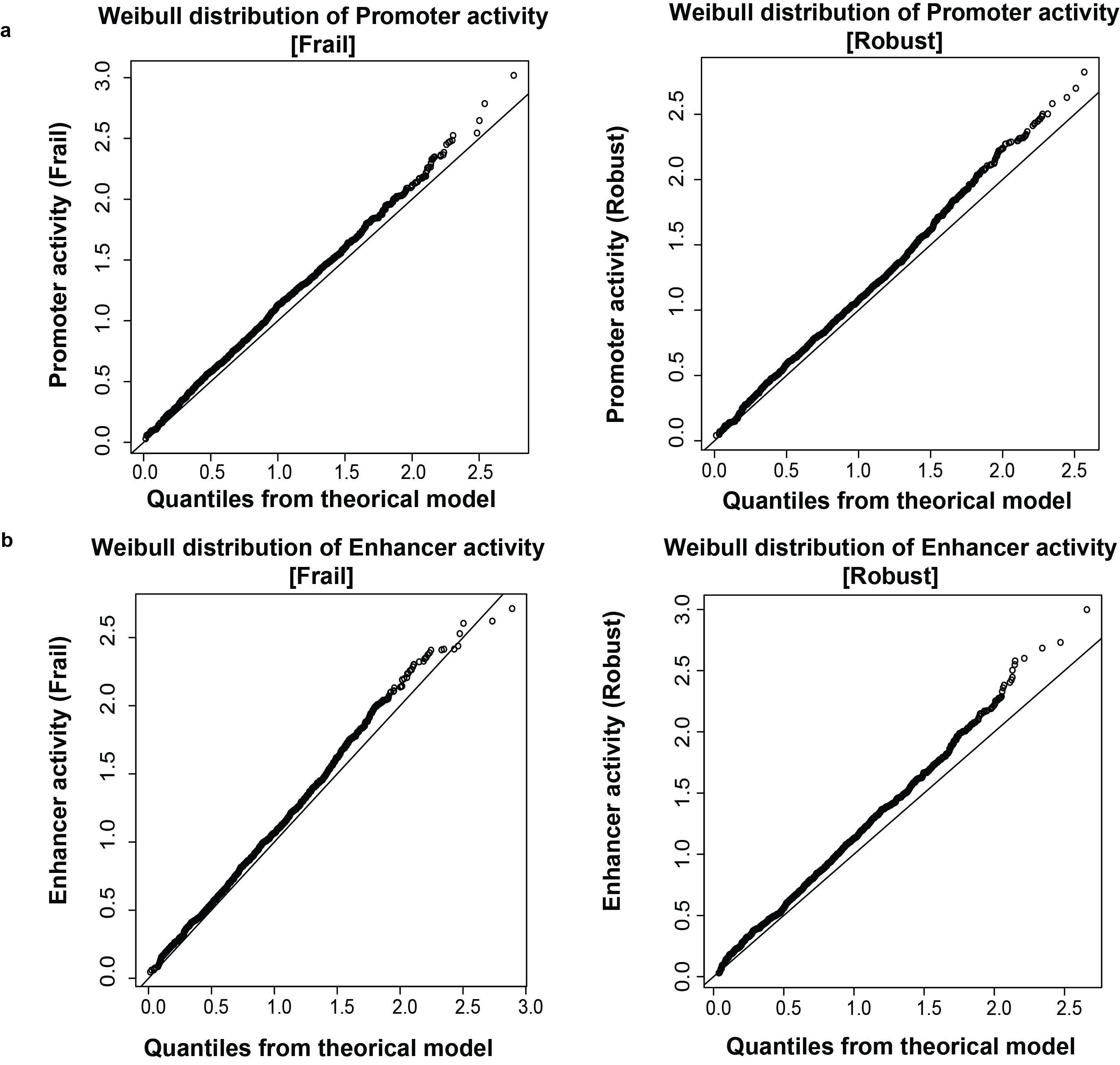

Supplement: Supplementary file 11 — (JPG 2674 kb) [file 11357_2023_961_MOESM11_ESM.jpg]
