# Supplementary material for: OVCH1 Antisense RNA 1 is differentially expressed between non-frail and frail old adults
Source: GeroScience. 2023 Oct 11;46(2):2063–81. doi: 10.1007/s11357-023-00961-9 (PMC10828349; doi:10.1007/s11357-023-00961-9)
Supplement: Supplementary file 12 — (DOCX 56.4 kb) [file 11357_2023_961_MOESM12_ESM.docx]

**Supplementary information (METHODS)**

OVCH1 Antisense RNA 1 is differentially expressed between non-frail and frail old adults

Imad Abugessaisa **^1*^**, Ri-Ichiroh Manabe **^2^**, Tsugumi Kawashima **^2^**, Michihira Tagami **^2^**, Chitose Takahashi **^2^**, Yasushi Okazaki **^2^**, Stefania Bandinelli **^3^**, Takeya Kasukawa **^1,4^**, and Luigi Ferrucci **^5^**

**^1^**Laboratory for Large-Scale Biomedical Data Technology, RIKEN Center for Integrative Medical Sciences, 1-7-22 Suehiro-cho, Tsurumi-ku, Yokohama City, Kanagawa, 230-0045, Japan.

**^2^**Laboratory for Comprehensive Genomic Analysis, RIKEN Center for Integrative Medical Sciences, 1-7-22 Suehiro-cho, Tsurumi-ku, Yokohama City, Kanagawa, 230-0045, Japan.

**^3^**AZIENDA USL TOSCANA CENTRO - InCHIANTI: Villa Margherita – Primo piano Viale Michelangelo, 41, 50125 Firenze, Italy

^4^Institute for Protein Research, Osaka University, Suita, Osaka 565-0871, Japan

**^5^**National Institute on Aging, National Institutes of Health, MedStar Harbor Hospital 5th floor, 3001 S. Hanover Street, Baltimore, MD 21225 USA.

*Correspondence

To whom correspondence should be addressed. Tel: +81 45 503 9111 (Ext 3467); Fax: +81 45 503 9604; Email: [imad.abugessaisa@riken.jp](mailto:imad.abugessaisa@riken.jp)

# RNA extraction, quality, and sequence library preparation

## Total RNA extraction

The quality of the extracted total RNA was measured using QIAxpert and processed the quality data with PAXgene (QIAGEN) RNA application. We observed the low A260:A230 values in several samples, this could due to the presence of guanidine thiocyanate, which was present in kit lysis buffers, used for RNA extraction. An additional purification step using the RNA Clean & Concentrator from Zymo Research was aimed at improving the quality of the RNA. The purification raised the A260:A230 ratio. RNA purification followed by RNA QC using NanoDrop.

## nAnT-iCAGE and LQ-ssCAGE library preparation, sequencing, and mapping

Three nAnT-iCAGE libraries (8-plex X 3) were prepared as described in[1]. In short, to include the 5`-end of the capped RNA first-strand, cDNA was transcribed and then CAGE barcode tags were attached. nAnT-iCAGE libraries were prepared before we depleted the globin (whole-blood samples are rich with β-globin mRNA read). Quant-iT™ PicoGreen™ dsDNA Assay Kits (ThermoFisher) and Agilent 2200 TapeStation system (Agilent) were used to check the quality of the resulting libraries. nAnT-iCAGE libraries were sequenced using Illumina HiSeq2500 platform with Single-Read read type and 50 base read length. Raw reads were quality checked by FastQC. The sequenced CAGE tags from all libraries were mapped to the human genome (hg38.p2) using STAR aligner. Sequences that match to rRNA reference sequences and sequences that have a low-quality base 'N' were removed.

After completing of the preparation of nAnT-iCAGE, we have enough amount RNA for 17 participants for preparation of LQ-ssCAGE. This enabled us to enrich the sequence profiles generated by nAnT-iCAGE (n=24). Prior to the preparation of LQ-ssCAGE, globin was depleted using GLOBINclear Kit (Thermo Fisher Scientific) followed by library preparation. LQ-ssCAGE started by reverse transcription to generate RNA/cDNA hybrids followed by CAP trapper procedures as described in [2]. The quantity of resulting libraries were checked by qPCR using KAPA Library Quantification Kits (Kapa Biosystems). LQ-ssCAGE library were sequenced using Illumina HiSeq2500 platform in Rapid mode with Paired-End read type and 50 base read length. FastQC was used to check the quality of the raw reads sequenced. The sequenced CAGE tags from LQ-ssCAGE library mapped to human genome (hg38.p2) using STAR aligner using the same setting as for mapping nAnT-iCAGE libraries.

# CTSS Annotation and basic processing

## Generation and post-processing of CTSS

As mentioned earlier, the nAnT-iCAGE libraries were prepared without depleting β-globin, this results in high level of β-globin mRNA in the mapped data, on the contrary for LQ-ssCAGE libraries we used GLOBINclear Kit (Thermo Fisher Scientific). β-globin mRNA introduces bias and masked lower abundance transcripts. Therefore, we masked β-globin mRNA from each sample (CTSS file) which reduced the number of the total tag counts in each CTSS. Furthermore, to avoid any possibility of presence of β-globin mRNA reads, we computationally retrieved all annotated β-globin mRNA from RefSeq database[3] and then using in-house custom script we filtered all defined β-globin mRNA regions from the CTSS files (**Supplementary Table 6**). Pooled CTSSs, active TSSs and active enhancers are merged and result in total of 65,441 merged clusters. The merged clusters are used for transcript annotation.

## Genomics annotation of CTSS

The CAGE method is specifically designed to profile the transcription start sites (TSS) and identify promoter regions. To confirm the quality of the CAGE profiles created by nAnT-iCAGE and LQ-ssCAGE, CTSS should be annotated to estimate the proportion of the promoter genomic regions. To annotate the CTSS we used CAGEr R Bioconductor package[4]. The first step in CAGEr was to create CAGEexp object. CAGEexp object created by specifying the genome assembly in BSgenome data package (BSgenome.Hsapiens.UCSC.hg38) and the location of the input files (list of CTSS files). To annotate the CTSS, annotateCTSS() CAGEr function was used. The input to the annotateCTSS was the CAGEexp object and the reference transcript model. We used the comprehensive gene annotation from GENCODE[5] (gencode.v37.annotation.gff3.gz). annotateCTSS intersect CTSS with the reference transcript model and identify four genomic regions (promoter, exon, intron and unknown).

## Distributions of CAGE tags per TSS

Typical CAGE dataset shown to follow a power-law distribution[6]. To verify that generated CTSS profiles followed the power-law distribution, we used plotReverseCumulatives () CAGEr function, which helps to specify the range values of the distributions of CAGE tags. The function takes as input the CAGEexp object which was created early and fitInRange which specifying a range of tag count values to be used for fitting a power-law distribution to reverse cumulative.

# Characteristics and quality of old adults’ CAGE profiles

## Promoter rich CAGE profiles follows power-law distribution.

The total number of the tag counts in CTSSs was 333,209,364. This number of tags contains the β-globin mRNA regions, after filtering the β-globin mRNA regions we retained total of 124,785,495 tag counts (TC) (37.4 % of the total tag counts) used for the rest of the analysis. The genomic annotation of the CTSS shows a promoter rich profile (**Supplementary Figure 1**) with 68.7% of the genomics regions annotated as promoter regions. Furthermore, the distributions of the CAGE profiles follow power-law distributions as shown in (**Supplementary Figure 2**). The usage (signal) of the CTSSs across all samples called pooled CTSSs, the quantification and normalization of the pooled CTSSs returned 21,781,984 CTSSs. The pooled CTSSs used for identifying TSSs and active enhancers.

## Quantification of CTSS, identification of the candidate uni- and bi-directional clusters

After confirming the quality of the CAGE profiles, the next step was to quantify the CTSS across all libraries (n=24). For this primary analysis, we used the R Bioconductor package CAEGfightR[7]. the BigWig files were inputed to the CAGEfightR functions, was. We used the function convertBED2BigWig() to convert the CTSSs files (in BED format) to BigWig files. Next was to quantify the CTSS usage across all libraries, here we used the function quantifyCTSSs(), which take as input the list of BigWig-files, the genome (sequence information) and optionally, a metadata information to be added to the CTSS-by-library count matrix. The resulting count matrix of the CTSS used for the CTSS level analysis. CTSS level analysis include the identification of the unidirectional TSSs candidates and the bidirectional clusters (enhancer candidates). CAGEfightR support CTSS level analysis by different functions. In short, CAGEfightR find the clusters by computing the pooled CTSS signal across all libraries. This is achieved by normalizing CTSSs raw count in each library to Tags-Per-Million (TPM), followed by sum-up the TPM values across all libraries. The function quickTSSs(), locate the uni-directional clusters (Tag clusters (TCs)) the candidates for TSSs. The majority of the identified TCs are lowly expressed and have little biological interest. We filtered out all TSS that did not have at least one TPM in at least 4 samples. The function quickEnhancers(), are used to identify bi-directional clusters (BCs) and take as input the pooled and normalized CTSSs. Similarly, to TSS we applied filtering to keep only BCs with at least 1 count in at least 4 samples.

## Cluster and gene level analysis

Once the biologically relevant and interesting TSSs and enhancers candidates are identified, the next step was to annotate them with transcript models. We used R Bioconductor annotation package TxDb.Hsapiens.UCSC.hg38.knownGene for the TxDb object used in CAGEfightR. For both the TSSs and the BCs annotation categories are promoter, proximal, fiveUTR, threeUTR, CDS, exon, intron, antisense and intergenic. For the BCs we kept the intergenic and intronic enhancers. The output from the cluster level analysis enabled us to investigate the shape and spatial characteristics of promoter. Prior to performing the differential TSS Usage (DTU)/ alternative TSSs and differential expression analyses, we performed annotation to the gene model by using the function assignGeneID() which annotate genomics ranges using the GeneBank ID. Finally, we quantified the number of TCs within the annotated genes by using quantifyGenes() function which sum-up the total number of TCs. At this stage the gene-level expression matrix was ready for the downstream analysis.

# Weighted gene co-expression network analysis and PPI

The input to WGCNA was the expression matrix obtained early, and the clinical data as reference outcomes. For the trait data we selected the following continuous variables age, HDL cholesterol (mg/dL), triglycerides (mg/dL), red blood cells (RBC)(n,millions/µL), hemoglobin (g/dL), hematocrit (%), blood glucose (mg/dL), blood urea nitrogen (mg/dL), serum creatinine (mg/dL), body mass index and two categorical variables sex (male vs. female), and frailty status (frail vs. robust). Following the creation of the expression and traits data, next step was to construct network and detect modules. WGCNA provides three methods to construct a network. We opted, to use the automatic network construction and module detection method. we used the recommended/default settings from WGCNA R package. We used the following code segment:

net = blockwiseModules(datExpr, power = 9,

TOMType = "signed", minModuleSize = 30,

reassignThreshold = 0, mergeCutHeight = 0.25,

numericLabels = TRUE, pamRespectsDendro = FALSE,

saveTOMs = TRUE)

The above code result in a gene network with 22 modules.

After identifying modules in the created network, we used two functions moduleTraitCor() and moduleTraitPvalue() to quantify module–trait association. Next, we quantify associations of individual genes in the expression data with all traits (clinical variables) by defining gene significance of the correlation between the gene and the clinical variables. For the individual module, WGCNA define a quantitative measure of module membership as the correlation of the module eigengene (ME) and the gene expression profile. Using the module membership variable as the ranking order, we performed an enrichment analysis using GSEA using anRichment R package. To identify the hub genes in the distinct module we exported the module gene to Cytoscape (3.9)[8] using the function exportNetworkToCytoscape() with threshold = 0.26. In Cytoscape we used the cytoHubba[9] plug-in to find and identify the hub genes (genes with high connectivity in the network). In Hubba nodes we identified the top 20 nodes ranked by degree. To look at the Protein-Protein-Interaction (PPI) network of the top hub genes we used STRING database (11.5)[10] to generate the PPI network.

## Enrichment of DNA-binding motifs

After identifying the differential expressed TSSs and active enhancers, we aim to predict the set of the transcription factors (TF) involved in the regulation of the TSSs and active enhancers. To answer to these questions, we annotate TSSs and active enhancers with the DNA-binding motifs from the JASPAR database[11]. Firstly, we extract the sequence around the TSSs and active enhancers by defining the region as +/- 500 bp around the TSS peak or enhancer midpoint. This result in an R DNAStringSet object. Secondly, we used the R package PWMEnrich.Hsapiens.background() and downloaded the pre-compiled background PWMLogn.hg19.MotifDb.Hsap. Thirdly, we used the function motifEnrichment() from R package PWMEnrich to scan the DNAStringSet object created early against the PWMEnrich.Hsapiens.background which result in MotifEnrichmentResults object. finally, we calculate motif enrichment scores in the MotifEnrichmentResults using the sequenceReport() which give a new object of type MotifEnrichmentReport which contains a table with five columns (Rank, Target (gene or Isoform), Motif-ID, raw score, and p.value).

## Motif activity calculation and analysis

CAGE data enable motif activity analysis. To calculate the motif activities, we utilized the methods described in[12, 13]. As input for the motif activity calculation, we prepared the list of promoters in BED format, expression data in tab-delimited format and a BED file containing the precalculated Transcription Factor Binding Site (TFBSs) which was provided by[13]. The actual calculation of the motif activities implemented in three steps. Firstly, calculate the binding profile of transcription factors (TF) with respect to the TSS, secondly, associate precalculated TFBSs with promoters / enhancers. Thirdly, calculate the motif activities under two conditions (Frail vs. Robust)

# REFERENCE

1. M. Murata, et al., “Detecting expressed genes using CAGE,” *Methods Mol Biol*, vol. 1164, 2014, pp. 67-85; DOI 10.1007/978-1-4939-0805-9_7.

2. H. Takahashi, et al., “Low Quantity Single Strand CAGE (LQ-ssCAGE) Maps Regulatory Enhancers and Promoters,” *Methods Mol Biol*, vol. 2351, 2021, pp. 67-90; DOI 10.1007/978-1-0716-1597-3_4.

3. W. Li, et al., “RefSeq: expanding the Prokaryotic Genome Annotation Pipeline reach with protein family model curation,” *Nucleic Acids Res*, vol. 49, no. D1, 2021, pp. D1020-D1028; DOI 10.1093/nar/gkaa1105.

4. V. Haberle, et al., “CAGEr: precise TSS data retrieval and high-resolution promoterome mining for integrative analyses,” *Nucleic Acids Res*, vol. 43, no. 8, 2015, pp. e51; DOI 10.1093/nar/gkv054.

5. A. Frankish, et al., “GENCODE reference annotation for the human and mouse genomes,” *Nucleic Acids Res*, vol. 47, no. D1, 2019, pp. D766-D773; DOI 10.1093/nar/gky955.

6. P.J. Balwierz, et al., “Methods for analyzing deep sequencing expression data: constructing the human and mouse promoterome with deepCAGE data,” *Genome Biol*, vol. 10, no. 7, 2009, pp. R79; DOI 10.1186/gb-2009-10-7-r79.

7. M. Thodberg and A. Sandelin, “A step-by-step guide to analyzing CAGE data using R/Bioconductor,” *F1000Res*, vol. 8, 2019, pp. 886; DOI 10.12688/f1000research.18456.1.

8. P. Shannon, et al., “Cytoscape: a software environment for integrated models of biomolecular interaction networks,” *Genome Res*, vol. 13, no. 11, 2003, pp. 2498-2504; DOI 10.1101/gr.1239303.

9. C.H. Chin, et al., “cytoHubba: identifying hub objects and sub-networks from complex interactome,” *BMC Syst Biol*, vol. 8 Suppl 4, 2014, pp. S11; DOI 10.1186/1752-0509-8-S4-S11.

10. D. Szklarczyk, et al., “The STRING database in 2021: customizable protein-protein networks, and functional characterization of user-uploaded gene/measurement sets,” *Nucleic Acids Res*, vol. 49, no. D1, 2021, pp. D605-D612; DOI 10.1093/nar/gkaa1074.

11. O. Fornes, et al., “JASPAR 2020: update of the open-access database of transcription factor binding profiles,” *Nucleic Acids Res*, vol. 48, no. D1, 2020, pp. D87-D92; DOI 10.1093/nar/gkz1001.

12. E. Arner, et al., “Transcribed enhancers lead waves of coordinated transcription in transitioning mammalian cells,” *Science*, vol. 347, no. 6225, 2015, pp. 1010-1014; DOI 10.1126/science.1259418.

13. T. Alam, et al., “Comparative transcriptomics of primary cells in vertebrates,” *Genome Res*, vol. 30, no. 7, 2020, pp. 951-961; DOI 10.1101/gr.255679.119.
